# Transfer learning improves pMHC kinetic stability and immunogenicity predictions

**DOI:** 10.1016/j.immuno.2023.100030

**Published:** 2023-12-21

**Authors:** Romanos Fasoulis, Mauricio Menegatti Rigo, Dinler Amaral Antunes, Georgios Paliouras, Lydia E. Kavraki

**Affiliations:** a Department of Computer Science, Rice University, 6100 Main St, Houston, 77005, TX, United States; b Department of Biology and Biochemistry, University of Houston, 4800 Calhoun Rd, Houston, 77004, TX, United States; c Institute of Informatics and Telecommunications, NCSR Demokritos, Patr. Gregoriou E and 27 Neapoleos St, Athens, 15341, Greece

**Keywords:** Transfer learning, Peptide-MHC, Machine learning, Peptide kinetic stability, Peptide immunogenicity

## Abstract

The cellular immune response comprises several processes, with the most notable ones being the binding of the peptide to the Major Histocompability Complex (MHC), the peptide-MHC (pMHC) presentation to the surface of the cell, and the recognition of the pMHC by the T-Cell Receptor. Identifying the most potent peptide targets for MHC binding, presentation and T-cell recognition is vital for developing peptide-based vaccines and T-cell-based immunotherapies. Data-driven tools that predict each of these steps have been developed, and the availability of mass spectrometry (MS) datasets has facilitated the development of accurate Machine Learning (ML) methods for class-I pMHC binding prediction. However, the accuracy of ML-based tools for pMHC kinetic stability prediction and peptide immunogenicity prediction is uncertain, as stability and immunogenicity datasets are not abundant. Here, we use transfer learning techniques to improve stability and immunogenicity predictions, by taking advantage of a large number of binding affinity and MS datasets. The resulting models, TLStab and TLImm, exhibit comparable or better performance than state-of-the-art approaches on different stability and immunogenicity test sets respectively. Our approach demonstrates the promise of learning from the task of peptide binding to improve predictions on downstream tasks. The source code of TLStab and TLImm is publicly available at https://github.com/KavrakiLab/TL-MHC.

## 1. Introduction

T-cell-based cellular immunity plays an important role in the adaptive immune system, by protecting the organism against pathological events [1]. One of the defense mechanisms involves the recognition, by T-cell receptors, of class-I Major Histocompatibility Complex (MHC) receptors loaded with non-self peptides [2]. In this context, an orchestrated balance between peptide-MHC (pMHC) Binding Affinity (BA), peptide stability, and immunogenicity is crucial for triggering an immune response. Consequently, designing computational tools that can predict which peptides bind to the MHC receptor, which pMHC complexes are stable enough to be presented on the surface of the cell, and which peptides can elicit an immune response, is crucial for designing therapeutics [3].

The quality and quantity of experimental pMHC BA measurements stored in public databases [4,5] has allowed computational approaches that predict pMHC binding to emerge early on [6–9]. The inclusion of Mass Spectrometry (MS) data has pushed the field of pMHC binding even further, and newly emerged Machine Learning (ML) models are more robust and accurate than ever before [10–12]. In contrast, the volume of pMHC kinetic stability data that exist in databases is much lower, possibly because of the low-throughput characteristics of the methods that are being employed for dissociation measurement [13]. The two most known tools for predicting pMHC stability are NetMHCstab and NetMHCstabpan [13,14]. In regards to detecting immunogenic peptides, the list of tools is much larger [15]. Nevertheless, similar to pMHC stability, good quality datasets of immunogenic peptides are by orders of magnitude smaller in size than their pMHC binding counterparts.

Focusing on the task of immunogenic peptide detection, many tools in the literature use BA/Eluted Ligand (EL) predictions as an important feature. The efficacy of using BA/EL for immunogenic peptide selection has been thoroughly discussed in the literature with experimental results to support it; Bjerregaard et al. [16] found that BA predictions on immunogenic neoepitopes (tumor epitopes with somatic mutations that make strong immunogenic candidates [17]) are significantly stronger than ones of non-immunogenic peptides. Similarly, Koşaloğlu-Yalçin et al. [18] suggested that BA predictions, along with length-rescaling, constitute the best feature for identifying neoepitopes from a pool of mutated peptides. The benchmarking study in [19] reported high Area Under Curve (AUC) results when using NetMHCpan4.0 [20] or MHCFlurry [21] for T-cell recognition in vaccinia virus-infected mice. Additionally, BA-derived measures involving the neoepitope and its corresponding wildtype, such as the differential agretopicity index, have shown to have predictive capabilities [22–24]. However, in more than one study, the use of differential agretopicity index was not shown to lead to better neoepitope recognition [18,25]. The study by Fritsch et al. [26] found that using netMHCpan2.4 results in accurate and rapid neoepitope identification, but also that the difference between the predicted BA of the mutated self and the self is not significant. In general, pMHC binding data alone is informative, but mostly not enough for accurate immunogenicity prediction [19,27,28].

The pMHC binding information has not only been used as a predictive feature in tools for immunogenic peptide prioritization, but has also been employed during the training process, specifically of models that predict immunogenicity by learning from small-in-size immunogenicity datasets. For instance, NetMHCstab and NetMHCstabpan have mixed stability predictions from their models with NetMHCCons [29] using a weighting factor *α* ranging between 0 and 1 that is fine-tuned to predict T-cell epitopes [13,14]. NetTepi [30] further explores this idea, by weighting BA predictions from NetMHCCons, stability predictions from NetMHCStab, and T-cell propensity predictions from the model described by Calis et al. [31] to predict T-cell epitopes. Similarly, previous work mixes an immunogenicity score predicted by a convolutional neural network that is trained on immunogenicity data, with BA and antigen processing transport efficiency, on the task of neoepitope prediction [32]. PRIME, a peptide immunogenicity predictor, uses EL predictions from a binding predictior as a feature during the learning process [33]. DeepHLApan employs a deep multitasking network that considers BA, MS, and immunogenicity data at the same time [34]. These approaches can be considered instances of knowledge transfer [35] that try to exploit the common elements between two related/correlated tasks. In this case, BA/EL predictions are being transferred as prior information to the downstream task of immunogenicity prediction.

Transfer learning, a form of knowledge transfer where a pre-trained deep learning architecture is fine-tuned on a task that is related to the original one, has seen much success [35]. On the pMHC binding prediction specifically, MHCnuggets used transfer learning on pretrained per-allele BA predictors to adjust to alleles with less peptide data and, also, to MS data [36]. Moreover, various methods have been using pre-trained protein language models [37,38] and fine-tuning them to pMHC data. BERTMHC uses pre-trained transformer models on protein sequences fine-tuned to MHC class-II binding data [39]. MHCRoBERTa [40] trains pan-specific MHC class-I BA predictors using a ROBERTa [41] pre-trained model. Finally, Gasser et al. [42] adapt BERTMHC to class-I MHCs and tries to provide a biological interpretation to the attention weights of the transformer.

In this study, we develop two transfer learning-based predictors for class-I pMHC stability (TLStab) and peptide immunogenicity (TLImm). Both TLStab and TLImm were created by pretraining a BA/EL predictor using BA and MS data, and this predictor was fine-tuned on pMHC stability data for TLStab, as well as peptide immunogenicity data for TLImm. To assess the effectiveness of fine-tuning BA predictors, we benchmarked different knowledge transfer approaches on the tasks of stability and immunogenicity that were previously proposed in the literature. Transfer learning is shown here to perform better than existing knowledge transfer approaches. TLStab exhibits state-of-the-art results in two different pMHC stability test sets. Finally, TLImm shows superior performance in a SARS-CoV-2 test set of pathogens [43]. TLStab and TLImm are both open-source and available at https://github.com/KavrakiLab/TL-MHC.

## 2. Materials and methods

### 2.1. Pretraining BA/EL predictors (TLBind)

To pretrain our BA/EL predictor, we acquired BA and MS datasets from [11] abd [44]. The training dataset consists of data from IEDB [4] (including both BA and MS data), with the addition of the MONOALLELIC MS dataset comprising 92 samples from HLAthena [45] and 8 samples from [46], as well as deconvoluted MULTIALLELIC MS datasets from various sources that were curated in and obtained from [11]. We further add decoys to the training dataset, that were generated from the same proteins that the positive peptides come from, as described in [11]. The training dataset was then split using stratified 5-fold cross-validation (CV), in order to select the hyperparameters that fit best the data, and we henceforth refer to this process as the *model selection* step. As soon as the model selection step was complete and the hyperparameters were chosen, the entire training dataset was used for training, with an extra validation set being used for early stopping. The MS part of the validation set was constructed from an additional MONOALLELIC dataset generated by [44]. We also included 15% of the BA data points from the IEDB that were not used during training, as well as a separate, smaller BA dataset that was acquired from [47]. In order to compare our pre-trained predictor to high-performing binding predictors found in the literature, a left-out test set was used. The left-out test set was constructed from an additional, separate to the previous ones, MULTIALLELIC dataset also used by [44]. Both validation and test sets were enriched with decoys generated from the human proteome, as described in [44]. We filtered out the pMHC pairs from the training dataset that were also found in the validation/test sets, in order to avoid data leakage during model evaluation. Overall, the training set consists of 164,582 BA datapoints and 482,720 MS hits (totaling ~2 million MS datapoints when decoys are considered), with the BA/MS ratio equaling to about 1:3 (~1:100 when MS decoys are considered). We will be referring to our pretrained BA/EL predictor as TLBind in the rest of the paper.

In terms of the class of models that were chosen, we follow conventions proposed by [8,11], and employed Multi-Layer Perceptrons (MLPs). Additional information in regards to model architecture, featurization and hyperparameter selection are provided in the Supplementary Methods found online.

### 2.2. TLStab

#### 2.2.1. Datasets

In order to train for pMHC stability prediction, the training dataset was acquired from the NetMHCstab study, totaling 6,298 data points from 10 different alleles, as described in [13]. We used 10-fold stratified CV to get an estimate on the model’s ability to generalize on the left-out test sets. Internally, for each training set created by the 10-fold CV, we performed a further 90%/10% train/validation split for hyperparameter tuning, as proposed in [48], leaving us with an ensemble of 10 MLPs. This way, no test samples from the 10-fold CV participated in hyperparameter tuning, resulting in an unbiased evaluation. To further test the method on datasets outside the training set distribution, a set of Ebola virus peptides [49] and a set of Pox virus peptides [50] were acquired from IEDB.

#### 2.2.2. Fine-tuning the BA/EL predictor

**Fig. 1.**
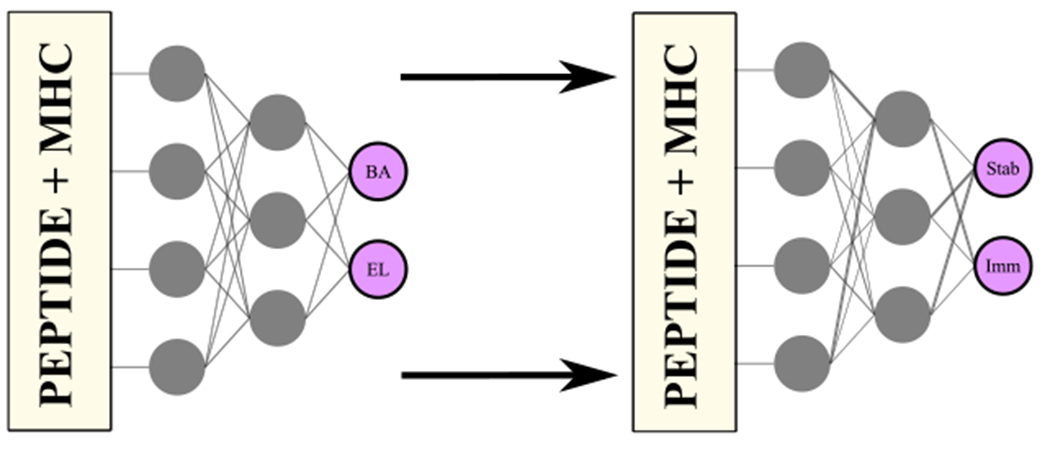
*TLStab & TLImm*: A BA/EL predictor similar to NetMHCpan4.1 [10] is fine-tuned to stability/immunogenicity tasks. This is achieved by refining the MLP weights through task-specific training.

TLStab was created by fine-tuning the pre-trained BA/EL predictor to the pMHC stability task. Specifically, during training, the MLP weights were loaded from our pre-trained BA predictor, instead of them being randomly initialized. The BA output of the network was then re-purposed for pMHC stability prediction, by exposing and fine-tuning the network to stability data (Fig. 1). During fine-tuning, we unfroze all the layers of the network and fine-tuned all the MLP weights to the new task. This was done due to earlier findings in regards to peptide amino-acid importances for pMHC stability; while it is known that the anchor positions of the peptide contribute the most to stability [13], newer findings have shown significant contributions of other peptide amino acid positions, for example, position 6 in HLA-A*02:01, visible in thermostability motifs [51]. By unfreezing all weights, we sought to alter the feature extraction stage, by slightly shifting the interest to those peptide positions too. For more information in regards to the datasets and methods for training TLStab, please refer to the Supplementary Methods found online.

### 2.3. TLImm

#### 2.3.1. Datasets

In order to train for peptide immunogenicity, the training dataset was collected from IEDB, with the filtering process being as described in [52], resulting in a dataset of 8971 points. We also incorporated 408 dengue virus positives from [53] into the training dataset. A stratified 5-fold CV process, similar to TLStab, was followed, both for model selection and evaluation. For model testing on out-of-distribution data, 92 viral SARS-CoV-2 peptides were obtained from [43], that were tested on convalescent/unexposed to SARS-CoV-2 donors. For testing TLImm on neoepitopes, we employed a filtered version of the neoepitope benchmark dataset from the Tumor neoepitope Selection Alliance (TESLA) consortium [24], containing 399 9-mer and 10-mer peptides.

#### 2.3.2. Fine-tuning the BA/EL predictor

Similar to TLStab, TLImm was initialized by loading the MLP weights of our pretrained BA/EL predictor. The EL output of the network was fine-tuned on immunogenicity data (Fig. 1). For similar reasons to TLStab, we also opted to unfreeze all the layers of the network; it is known that, for pMHC binding, it is the anchor positions of the peptide that mediate the peptide-MHC interaction, namely position 2 and the C-terminus positions [54,55]. However, for peptide immunogenicity, it is the amino-acids in the middle portion of the peptide that are mostly coming in contact with the T-cell receptor [31,52,56]. By unfreezing all the layers, we sought to slightly alter the neural network weights that correspond to the middle portion of the peptide. For information on dataset filtering and explanatory analysis for the task of peptide immunogenicity, please refer to the Supplementary Methods found online.

## 3. Results

### 3.1. Correlation of BA values/predictions to pMHC kinetic stability/immunogenicity

**Fig. 2.**
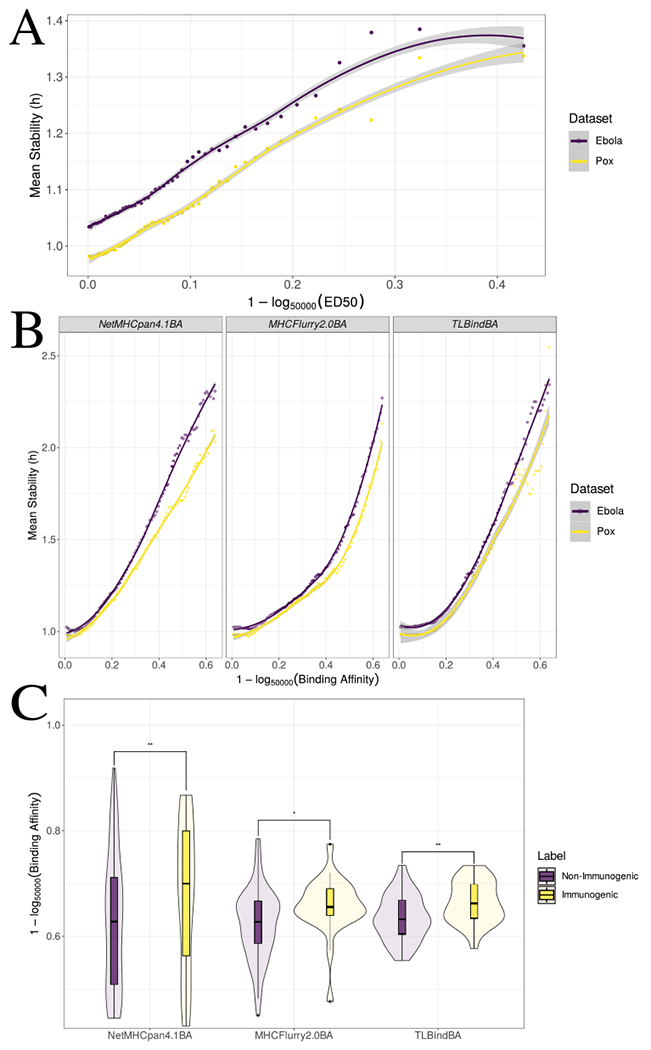
(A) Relationship between experimental ED50 values and stability values on the Ebola virus dataset and the Pox virus dataset. The *y*-axis depicts the mean stability values of peptides that have a better ED50 than the threshold (x-axis). (B) Relationship between BA predictions from two state-of-the-art tools (plus our pre-trained BA/EL predictor TLBind) and stability values. The *y*-axis depicts the mean stability values of peptides that have a better predicted BA than the threshold (x-axis). (C) NetMHCpan4.1 (*p* < 0.001), MHCFlurry2.0 (*p* < 0.01) and TLBind (*p* < 0.001) affinity predictions on immunogenic peptides are significantly different when compared to non-immunogenic ones.

It has already been reported in the literature, albeit in small datasets, that there is a significant correlation between binding affinity and stability/immunogenicity [16,18,19,57]. We wanted to assess the validity of this hypothesis in other publicly available datasets. Validating this hypothesis motivates the use of knowledge transfer, particularly transfer learning methodologies, for the tasks of peptide stability and immunogenicity prediction. We acquired the three largest publicly available stability datasets currently found in IEDB, namely, the training dataset from the NetMHCstab study [13], a dataset of Ebola virus peptides [49] and a dataset of Pox virus peptides [50] (see Supplementary Methods for more information on the datasets). In Fig. 2A the relationship between experimental ED50 values provided in the Ebola and Pox virus datasets and pMHC stability is shown. Specifically, for different ED50 thresholds, we calculate the average stability value (y-axis) of the peptides that exhibit a better ED50 value than a selected ED50 threshold (x-axis). Higher ED50 thresholds correlate with higher mean stability values, showing that affinity values can be used to infer pMHC stability.

This is not true solely for experimental values, but also for BA/EL prediction tools. Specifically, we acquired BA predictions from NetMHCpan4.1, MHCFlurry2.0 and TLBind, our pre-trained BA predictor that exhibits similar performance with state-of-the-art tools on the task of binding prediction (Supplementary Table S1). BA predictions were taken for both the Ebola virus and Pox virus datasets. Again, there is a monotonic relationship between BA predictions and the pMHC mean stability values (Fig. 2B). The same relationship can be seen when NetMHCpan4.1, MHCFlurry2.0 and TLBind are used for the NetMHCstab dataset (Supplementary Figure S1). This hints at an important realization; specifically, that there is potential in harnessing properties of BA prediction tools and adjusting them downstream in order to predict peptide kinetic stability. In other words, BA prediction tools seem to be a good starting point for this particular task. It is worth underlining here that this is not a property stemming from BA data only, but also MS data. This is important, as the training set of TLBind consists primarily of MS datapoints (see Methods), therefore, correlation between MS outputs and stability should also be present, in order to get the most out of the pMHC binding datasets. Indeed, the EL/presentation score outputs of the aforementioned tools, trained on big MS datasets, also correlate with peptide stability in the same way as BA predictions do (Supplementary Figure S2). Therefore, there is potential for transfer learning methodologies to be applied on state-of-the-art BA/EL predictors, which benefit from both big BA and MS datasets, so that these tools are re-purposed for peptide stability prediction.

It is worth stressing here that the relationship between BA and stability is better seen when the mean stability of a pool of peptides is calculated (Fig. 2, Supplementary Figure S1); by plotting a 1:1 relationship between affinity and stability, there are many datapoints which do not follow this trend. Specifically, emphasizing on the NetMHCstab training dataset, there are strong binders with low stability (as previously seen in [57]), but also seemingly weak binders with much better stability values than the strong ones (Supplementary Figure S3). The latter cases have been observed more scarcely, but still seen in the literature. For instance, peptide mutations in anchor positions that result to better BA values have shown to make the peptide more unstable than its wildtype counterpart [58,59]. Regarding BA predictors, these cases could just be false predictions, however, this trend is true even when looking at measured ED50 values in the Ebola and Pox virus datasets (Supplementary Figures S4). This indicates that BA/EL prediction tools alone are not sufficient for peptide stability prediction, and adjustment to peptide stability datasets is necessary.

Similar conclusions can be derived by observing the relationship between BA predictions and immunogenic/non-immunogenic labels. Distributions of BA predictions by NetMHCpan4.1, MHCFlurry2.0 and TLBind for the two different classes of immunogenic/non-immunogenic peptides for the SARS-Cov-2 viral dataset [43] can be seen in Fig. 2C. We removed peptides from the analysis that were not predicted to be strong binders, and thus, possibly non-immunogenic (predicted BA > 500 nM), in order not to bias towards possible non-binders existing in the dataset. Using the Kolmogorov-Smirnov non-parametric test, we confirmed that the distributions are significantly different for NetMHCpan4.1 (*p* < 0.001), MHCFlurry2.0 (*p* < 0.01) and TLBind (*p* < 0.001), reinforcing the hypothesis that BA prediction models/values can be used to discriminate between immunogenic/non-immunogenic peptides, and therefore, can be adjusted using knowledge transfer approaches to the downstream task of peptide immunogenicity prediction. Similar results are obtained when comparing the distributions of BA predictions in the IEDB dataset that was used to train TLImm (Supplementary Figure S5).

### 3.2. Performance of TLStab

**Fig. 3.**
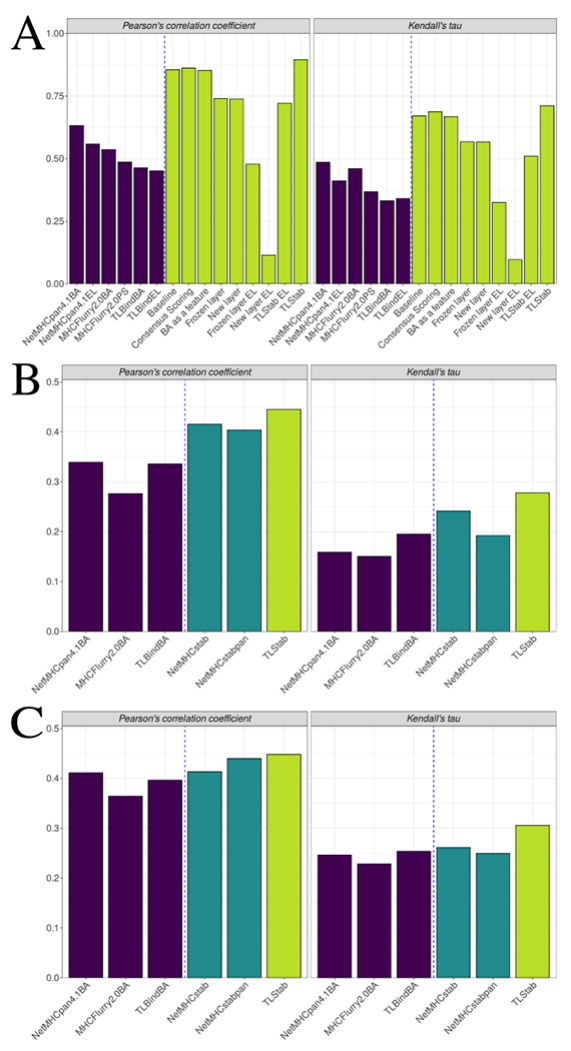
(A) Pearson’s correlation and Kendall’s tau performance of TLStab against other knowledge transfer approaches on the unbiased 10-fold nested CV experiment. On the left part of the blue dashed line, the performance of BA/EL predictors is depicted (blue bars). On the right side, we show the performance of various knowledge transfer approaches and TLStab (dark yellow bars). (B) Pearson’s correlation and Kendall’s tau performance of TLStab against other approaches on the Ebola virus Dataset. On the left part of the blue dashed line, the performance of BA predictors is depicted (blue bars). On the right side, we show the performance of state-of-the-art pMHC stability tools (teal bars) compared to TLStab (dark yellow bar). (C) Pearson’s correlation and Kendall’s tau performance on the Pox virus Dataset. (For interpretation of the references to color in this figure legend, the reader is referred to the web version of this article.)

As a first experiment, we wanted to assess whether transfer learning is the most predictive knowledge transfer approach. We benchmarked TLStab (see Methods and Supplementary Methods for details on the training process to create TLStab) against different knowledge transfer approaches that have been previously proposed in the literature [13,33,52], in order to select the most predictive one for the task in an unbiased way (see Supplementary Methods for a detailed description of the different architectures that were used for benchmarking). For each knowledge transfer approach, we performed a 10-fold stratified cross validation process (discussed in Methods), and subsequently performed model evaluation on the 10 left-out test datasets. For model evaluation, we employed Pearson’s correlation coefficient and Kendall’s tau-b, as proposed in [21]. In the unbiased 10-fold scenario evaluation, the commonly accepted way of reporting results is the mean performance of the 10 folds, accompanied by the standard deviation [48]. However, correlation coefficients are notorious when averaging, as their sampling distribution is skewed, leading to underestimation. Moreover, applying the known Fisher’s *z*-transform before averaging leads in overestimation [60]. Thus, we merged the predictions and the labels of the 10 left-out test sets into one large test set, as described in [61]. The performance of the different knowledge transfer techniques (with the inclusion of NetMHCpan4.1, MHCFlurry2.0 and TLBind BA/EL predictions as further baselines) can be seen in Fig. 3A . TLStab led to better performance than all other methods, an indication that, initializing a predictor’s weights with BA information and fine-tuning those weights to stability data leads to better generalization. It is important to note that, although the EL output of the binding prediction tools exhibits fair correlation with the stability levels, the BA output exhibits even higher correlation. This also affects the fine-tuning process, where it can be seen that finetuning on the BA output instead of the EL output results in a better correlation. Lastly, we observed that unfreezing all the neural network weights results in a better performance. As the middle portion of the peptide has proven to affect peptide stability [51], we hypothesized that the feature extraction step of the first layers also needs to be fine-tuned. This hypothesis was confirmed experimentally. Lastly, all the above results hold true even when we examine the data on a per-allele basis (Supplementary Figure S6).

We also benchmarked the performance of TLStab against other state-of-the-art approaches on the Pox virus [50] and Ebola virus peptides [49]. We filtered out the HLA-B*08:01 allele datapoints from the two datasets, so that the two datasets only contain alleles suported by NetMHCstab – an allele-specific method – for comparison purposes. This resulted in 978 data points for the Ebola virus dataset and 522 data points for the Pox virus dataset. For each pMHC instance, we predicted stability with TLStab by averaging the 10 predictions from each MLP in the 10-model ensemble. We also included in the comparison the BA predictions of NetMHCpan4.1, MHCFlurry2.0 and TLBind. In the Ebola virus dataset, TLStab performs better than all other methods both in terms of Pearson’s correlation coefficient and Kendall’s tau (Fig. 3B). The results are similar in the Pox virus dataset, where, although Pearson’s correlation is similar to NetMHCstabpan, TLStab achieves evidently better ranking performance against all other methods (Fig. 3C).

A common characteristic of NetMHCstab, NetMHCstabpan and TLStab is that they were trained on datasets where all non-negative instances are strong binders, as peptide binding is a requirement for measuring stability when performing a scintillation proximity assay [13]. Specifically, NetMHCstabpan reports that all positive instances have either BA < 500 nM or %2 rank [14]. To balance the datasets in terms of unstable peptides, both NetMHCstab and NetMHCstabpan sample random negatives (BA > 20000 nM) that are labeled with 0 half-life labels. Nevertheless, the non-zero half-life datapoints are all strong binders. As such, we also wanted to evaluate the models on a dataset that only contained strong binders. We filtered the Ebola and Pox datasets, so that BA predictions given by MHCFlurry2.0 were lower than < 500 nM to simulate the training data conditions. The filtered Ebola and Pox datasets contain 321 and 185 datapoints respectively, all predicted to be strong binders by MHCFlurry2.0. For model testing, Pearson’s correlation coefficient and Kendall’s tau-b was again used. Results are shown in Supplementary Figure S7. It is notable that most methods benefit from the strong binder filtering, most likely due to the fact that their training dataset comprises strong binders + non-binders, excluding weak binders. TLStab outperforms all other approaches for both metrics in the Ebola virus dataset. Interestingly, all stability predictors on the Pox Virus dataset do worse than binding affinity predictors. We believe this to be an artifact, as the number of data points left in the Pox virus dataset after the filtering is quite low in comparison to the full dataset.

We also compared our method against NetMHCstabpan in the Ebola virus and the Pox virus datasets containing the allele HLA-B*08:01, which is not supported by NetMHCstab. In most of the cases, TLStab performs comparably, or outperforms NetMHCstabpan in terms of ranking (Supplementary Table S2), although the number of HLA-B*08:01 datapoints in both Ebola and Pox datasets is small in relation to the full datasets (45 HLA-B*08:01 points for the Ebola virus and 19 HLA-B*08:01 data points for the Pox virus dataset respectively). As such, a more extensive dataset is needed to fully compare with NetMHCstabpan on out-of-training-distribution alleles. Furthermore, we benchmarked TLStab against other knowledge transfer approaches (see Supplementary Methods) for a more extensive comparison. In most of the datasets, the best results are achieved either by TLStab or the simpler “BA as a feature” model (Supplementary Table S2). Lastly, we also observe that fine-tuning on the EL output does not produce the same level of correlation as fine-tuning on the BA output (Supplementary Table S2).

### 3.3. Analysis of stability motifs

Similar to [13,51], we wanted to assess the difference between BA motifs and stability motifs, in order to interpret what is being learned by each model. We focused on 9-mers and the 10 alleles of the NetMHCstab training dataset. We extracted 500,000 9-mers by fragmenting protein sequences from the human proteome. We paired those peptides to each of the 10 alleles, and we used NetMHCpan4.1BA, NetMHCstab, NetMHCstabpan and TLStab to score the peptides for each allele. We used the scores of each tool to rank the peptides, and, by taking the top 0.1% (equal to 500 peptides per allele), we generated the peptide motifs for each tool. To generate and visualize the motifs, we used motifStack [62].

**Fig. 4.**
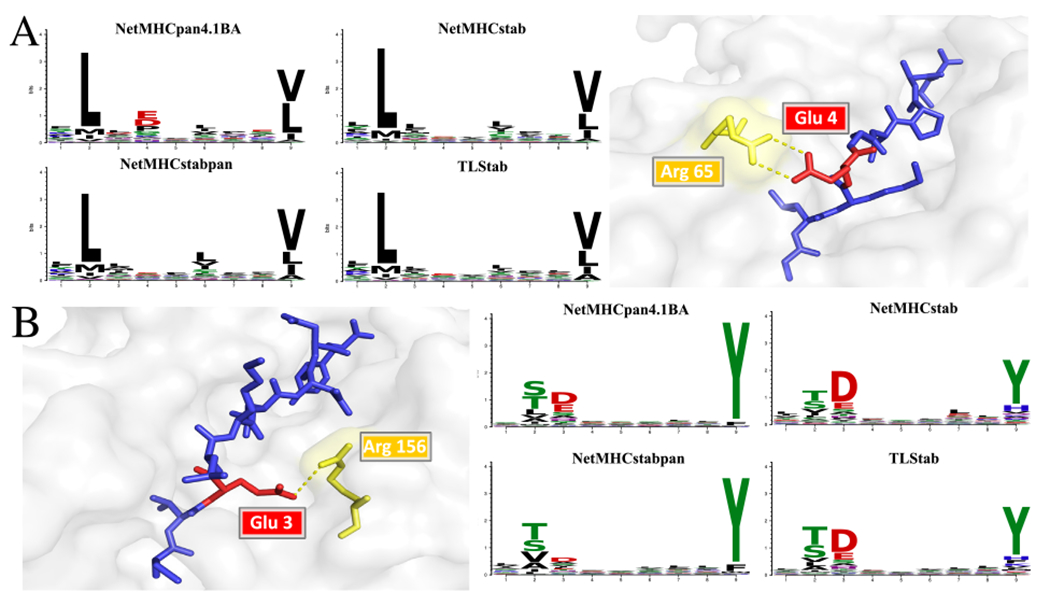
(A) HLA-A*02:01 motifs for the four depicted methods. While the binding affinity predictor has visible presence of negative charge in position 4, the stability prediction methods are less enriched, although the contribution of negative charge to peptide stability has been previously studied [63]. (B) HLA-A*01:01 motifs for the four depicted methods. The NetMHCstabpan motif has substantially lower presence of D3 and E3, although there is experimental evidence of formed bonds that contribute to peptide stability.

The complete list of motifs can be seen in Supplementary Figure S8. We observe the same enrichments in position 1 and position 3 in the majority of alleles for the stability methods as also reported in [13]. Additionally, all stability motifs agree to a great extent. However, there are evident differences between some stability and BA motifs, also observed in [51]. Such case was the allele HLA-A*02:01. For NetMHCpan4.1BA, a binding affinity predictor, the existence of negative charge in position 4 is important for good binding. However, the same is not true for stability predictors, where the enrichment in position 4 in regards to negative charge is much lower. This is contrary to recent works emphasizing the importance that formed salt bridges between position 4 and HLA-A*02:01 positions R65-K66 have on peptide stability [63]. The salt bridge formation is also evident on one example structure found in the PDB (PDB code: *5ENW*), where peptide GLKEGIPAL is bound to HLA-A*02:01 (Fig. 4A). We hypothesize that the reason for this low enrichment in position 4 across all stability tools might be the relatively small HLA-A*02:01 stability datasets that were used for training. These datasets might not contain statistically sufficient data exhibiting this relationship. However, previous work also suggests that the existence of negative charge might not necessarily correlate with enhanced pMHC complex stability. For example, in [64], the natural occurring mutation G4E on the known influenza peptide GILGFVFTL is not shown to affect pMHC complex stability. Additionally, according to [65], different MAGE-A variants, most of them exhibiting a D4, are less stable than the influenza or the tax peptide that both exhibit G instead. These results indicate that the existence of negative charge in position 4 might not be a prerequisite for peptide stability. However, more extensive studies need to be performed to determine the contribution of D4 and E4 to peptide stability for peptides bound to HLA-A*02:01.

There is also a visible motif difference between different stability methods in the case of the HLA-A*01:01. Specifically, there is a comparable existence of negative charge in position 3 in the NetMHCstab and TLStab motifs in comparison to NetMHCpan4.1BA (Fig. 4B). However, NetMHCstabpan is less conserved in position 3, while the glutamic acid is visibly less present. There are two structures deposited in Protein Data Bank (PDB codes: *4NQX, 4NQV*) with presence of E3 peptides bound to HLA-A*01:01. As reported in [66], in both structures, E3 is partially buried in the D pocket of the HLA-A*01:01 and forms a salt bridge with the R156, which hypothetically contributes to stability (Fig. 4B). The seemingly increased tolerance of NetMHCstabpan in position 3, as seen in the HLA-A*01:01 stability motif, could be also related to the reportedly worse performance for HLA-A*01:01 in the Ebola and Pox virus datasets (Supplementary Figure S9). Specifically, we found cases where NetMHCstabpan clearly overestimates stability of peptides not exhibiting an D3 or an E3.

### 3.4. Performance of TLImm

Given the good results of TLStab on the stability prediction task, the same fine-tuning idea was applied on the task of peptide immunogenicity prediction. The immunogenicity assays dataset was obtained from IEDB, following the filtering process previously proposed in the literature [52]. The additional Dengue virus pathogens [53] that were used in [52] as a validation dataset were instead added to the main dataset, since model selection was done through a 80%/20% split of the training set in each of the five folds.

Same as TLStab, we wanted to first assess if transfer learning is the most appropriate knowledge transfer approach for the task of peptide immunogenicity prediction. We performed 5-fold stratified CV for model selection and evaluation. Results can be seen in Supplementary Table S3. BA/EL predictors can distinguish between immunogenic/non-immunogenic peptides better than random. Their AUC values are all larger than 0.5 (the baseline AUC), and all Area Under Precision-Recall Curve (AUPRC) values are also above the baseline (fraction of positive instances in the dataset = 0.476). TLImm performs clearly better in comparison to other knowledge transfer approaches in terms of both AUC and AUPRC (Supplementary Table S3). Moreover, when comparing to freezing/replacing layers, finetuning all the network weights results in a better fit. This is consistent with the idea that the weights connected to the middle portion of the peptide need to be slightly adjusted, as it is this portion of the peptide that is interacting with the T-cell receptor [31,52,56]. Additionally, contrary to the peptide stability prediction task, fine-tuning on the EL output of TLBind results in a slightly better AUC and AUPRC values than when fine-tuning on the BA output. This is in agreement with previous reports indicating that the EL/presentation score output of binding prediction tools is superior to the BA output [28]. Lastly, as it has been previously observed that class-I pMHC stability is a better predictor than BA [57], we also performed fine-tuning on the peptide immunogenicity task using the weights of TLStab instead. Nested CV results were comparable with TLImmBA, and still inferior to TLImmEL.

**Fig. 5.**
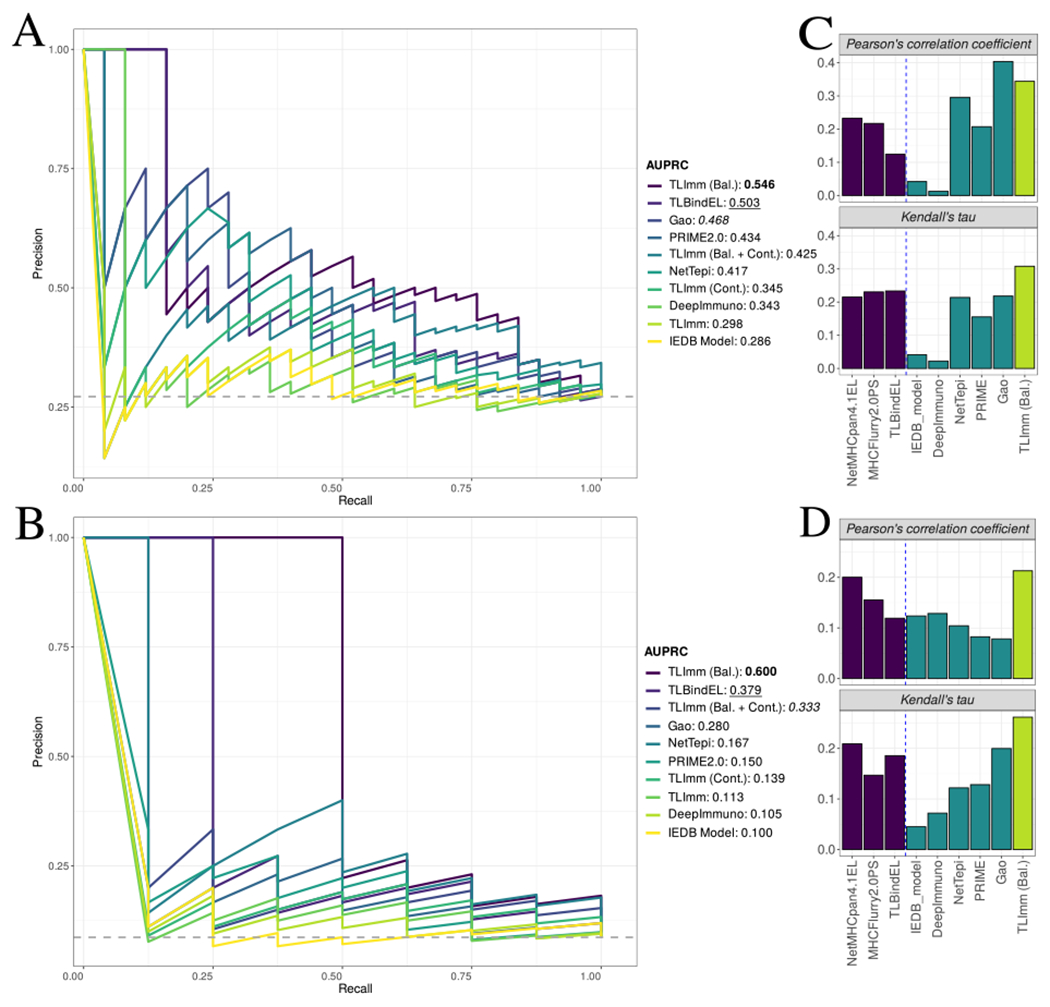
(A) *AUPRC* performances of different approaches on the **convalescent** donor labels. Baseline AUPRC Is depicted In dashed gray. (B) *AUPRC* performances of different approaches on the **unexposed** donor labels. (C) *Pearson’s correlation and Kendall’s tau* performances of different approaches on the response frequencies of **convalescent** donors. (D) *Pearson’s correlation and Kendall’s tau* performances of different approaches on the response frequencies of **unexposed** donors.

To compare TLImm against other peptide immunogenicity predictors in the literature, we used a dataset comprising a set of viral SARS-CoV-2 peptides. Specifically, the dataset contains 92 SARS-CoV-2 peptides that were tested either on convalescent or unexposed to SARS-CoV-2 donors [43]. From a computational perspective, this means that 2 immunogenic labels are associated with each pMHC instance: one coming from convalescent and one from unexposed donors. There are 25 positive instances related to convalescent donors, and 8 positive instances related to unexposed donors. As the negative class outnumbers the positive class, AUPRC is the preferred measure of performance over AUC [67]. The AUPRC performance compared to a list of other peptide immunogenicity prediction tools [30,31,52,68,69], both for convalescent and unexposed donor labels, can be seen on Fig. 5A and 5B, respectively. TLImm performs similarly to DeepImmuno in both convalescent and unexposed donors, which is expected, due to the use of the same training set. However, with the sole exception of the IEDB Model [31], the performance of TLImm seems to be lower than the performance of many other immunogenicity prediction tools. Moreover, TLBindEL, our pretrained baseline EL predictor, performs comparatively better than TLImm in terms of AUPRC, which was unexpected given that TLBind had not seen any immunogenicity data to begin with.

Given these results, we sought to understand how fine-tuning in the immunogenicity dataset actually worsens the performance of our predictor. We subsequently identified two areas of possible experimentation, both dealing with modifying the training set of TLImm:

1. *Data Labeling*: We experimented with labeling datapoints in our training dataset with either binary (indicating immunogenic/non-immunogenic status) or continuous, between 0–1 (indicating immunogenic strength) labels [52].
2. *Per-allele Balancing*: We subsampled datapoints of the training set with the purpose of equalizing the label balance for all alleles.

For more information/analysis on the effects of balancing and labeling the immunogenicity dataset, please refer to the Supplementary Methods found online.

Four combinations of TLImm arise from the above processes:

1. *TLImm*, trained on an imbalanced dataset with binary labels.
2. *TLImm* + *Bal.*, trained on a balanced dataset with binary labels.
3. *TLImm* + *Cont.*, trained on an imbalanced dataset with continuous immunogenic strength labels.
4. *TLImm* + *Bal.* + *Cont.*, trained on a balanced dataset with continuous immunogenic strength labels.

The four variations of TLImm were subsequently tested on the SARS-Cov-2 dataset. The first observation is that, by training on a dataset that is balanced, the proposed method outperforms all other approaches, including TLBindEL. That is true in both convalescent and unexposed donor labels. This shows that fine-tuning a pre-trained binding affinity predictor on a smaller dataset of viral peptides can result in a peptide immunogenicity model that performs better in out-of-distribution datasets of viral peptides, as long as the training dataset is free of any per-allele bias. Interestingly, incorporating continuous immunogenic strength labels does not result in better prediction. This is unexpected, as, in theory, more fine-grained information about the immunogenic strength of the peptide should result in the model being more accurate in its predictions.

Apart from the binary classification task, we also wanted to assess whether TLImm predictions correlate with recognition frequencies (number of positive responses in donors divided by total number of tested donors). In this way, we wanted to investigate the model’s ability to rank more potent peptides in terms of recognition frequency than less potent/non-immunogenic ones. Pearson’s correlation coefficient and Kendall’s tau results for both convalescent and unexposed patients are shown in Fig. 5C and 5D, respectively. Focusing on the convalescent donors, while the model from Gao et al. [69] exhibits better Pearson’s Correlation Coefficient, TLImm performs better with respect to ranking the more potent peptides in terms of recognition frequencies. Similar results can be observed in regards to the unexposed donors; in regards to Pearson’s correlation coefficient, TLImm performs similarly to NetMHCpan4.1EL, however TLImm still ranks higher the most potent peptides in terms of recognition frequencies.

The full benchmark analysis, incorporating BA/EL predictors and peptide stability predictors can be found in (Supplementary Table S4). The balanced version of TLImm outperforms all BA/EL predictors and stability predictors in both convalescent and unexposed donors, when considering either binary labels or continuous recognition frequency labels. Additionally, we wanted to compare TLImm against other knowledge transfer approaches. The results of this benchmark can be found in (Supplementary Table S5). TLImm outperforms all other knowledge transfer approaches for all combinations of datasets (convalescent/unexposed) and metrics (AUPRC/Kendall’s tau), at least when considering the balanced version of the training dataset, which gives the optimal results overall. Furthermore, similar to the nested CV experiment, the EL output of TLBind, when fine-tuned, achieves better results than when the BA output of TLBind is fine-tuned. Finally, as the nested CV already indicated (Supplementary Table S3), finetuning the weights of TLStab and the stability output does not lead to better results than those of TLImmEL.

In addition to viral peptide immunogenicity prediction, we assessed the performance of TLImm on a set of neoepitopes. For this experiment, we used the TESLA [24] neoepitope dataset, applying the same filtering methods as described in [28], therefore keeping 27 immunogenic neoepitopes and 372 non-immunogenic instances. AUC and AUPRC scores for all methods were calculated following the protocols described in [28] for a fair comparison. We also used Top-20 and Top-50 scores as previously described [24,32,52]. These thresholds reflect the number of peptides that are included in a personalized treatment [24]. The performance of TLImm can be seen in (Supplementary Table S6). Except the model from Gao et al. [69], TLImm outperforms all other immunogenicity prediction tools. However, even the balanced version of TLImm is ouperformed by BA/EL predictors, most notable TLBindEL, which exhibits the best performance across all tools. This is in agreement with previous findings [27,28], stating that pathogenic peptides features, such as dissmilarity-to-self, are innately different than features from neoepitopes. As a result, it is challenging for models trained on pathogenic datasets to generalize on neoepitope datasets [27]. Future work will include repeating the training and testing process of TLImm to datasets explicitly composed of neoepitopes, in order to further improve performance on the task of neoepitope identification.

## 4. Discussion

To elicit an adaptive immune response, epitopes undergo a series of intracellular processes happening in a sequential manner [57]. There are three main processes that have previously received great interest in the literature: (a) The binding of peptides to the MHC-I receptor, (b) the stability of the pMHC complex, and (c) the recognition of the pMHC by the T-cell receptor. The advent of MS has shed light on the pMHC binding and presentation process, and pMHC BA/EL prediction models [10,11,45] have greatly improved their accuracy on benchmark pMHC datasets. However, the amount of pMHC stability and immunogenicity data is much lower than BA/EL, and the landscape of both pMHC stability and immunogenicity prediction is largely underexplored.

To our knowledge, the most used pMHC stability predictors are NetMHCstab and NetMHCstabpan [13,14]. One of the reasons that the field of pMHC stability prediction has been insofar underdeveloped is the low-throughput of dissociation measurement experiments [13]. However, while not perfect, there is a measurable correlation between affinity and stability measurements that, although previously observed [57], it has not been considered as a way to account for the lack of stability data (Fig. 2A). This relationship should be exploited, as pMHC stability predictions have shown to be of invaluable use; pMHC stability has proven to be an important factor for T-cell recognition, both in previous work [13,14,30,57], as well as in the recent TESLA global consortium benchmark study on neoepitope identification [24]. We benchmarked different knowledge transfer approaches inspired from the literature, and found that transfer learning can help in improving pMHC stability prediction in the absence of abundant stability data (Fig. 3A). Furthermore, the proposed method, TLStab, improves upon the performance of previously established pMHC stability prediction tools, showcasing the effectiveness of fine-tuning BA predictors on stability data (Fig. 3B–C).

Likewise, the size of immunogenicity datasets still does not compare with the size of pMHC binding data. However, contrary to the pMHC stability tools, immunogenicity prediction tools are many more in number, especially when focusing on neoepitope identification [15]. These tools employ pMHC binding prediction and ML tools (where BA/EL are important features) to guide their epitope selection. This central theme of using BA/EL data/predictions for epitope selection stems from the bibliography that highlights an inherent relationship between pMHC BA/EL predictions and T-cell recognition [16,18,26,34,52,68]. We benchmarked different knowledge transfer approaches to validate which of these approaches is suited for immunogenicity prediction. Once more, transfer learning exhibits a greater potential in generalization (Supplementary Table S3). Additionally, the proposed TLImm method outperforms all other methods in identifying SARS-Cov-2 viral peptides (Fig. 5 and Supplementary Table S4). These results showcase the potential of transfer learning for viral peptide immunogenicity prediction.

We additionally highlight that, while the ML model and architecture selection are vital to the final performance obtained, data curation and filtering are also vital for building a robust model [70]. In particular, one needs to (a) ensure that labels are representative of the data points, and (b) there is sufficient label balance across different existing subgroups of the dataset in order to avoid potential bias [71]. In the pMHC immunogenicity prediction setting, this translates to (a) the immunogenicity label being representative of the pMHC pair, and (b) balance between immunogenic/non-immunogenic labels per allele. The study presented in [28] showed that there is per-allele label imbalance in the epitope datasets that are being employed, and, by benchmarking different peptide immunogenicity predictors, highlighted that this affects the performance of these models. We took this analysis a step further, and showed that, when the ML architecture takes into account the peptide sequence and the MHC sequence [32,52] , the model mostly identifies the type of allele in question, and not key properties of the peptide sequence (see Supplementary Methods for the full analysis). We subsequently showed that per-allele balancing of the training set can lead to better generalization performance (Fig. 5 and Supplementary Table S4).

Admittedly, our study has some limitations. Although TLStab outperforms state-of-the-art approaches, it does not achieve high enough correlation to experimental stability values. Using the biostatistical criteria by [72,73], in both out-of-distribution test sets (Pox virus and Ebola virus peptides), TLStab exhibits fair correlation at best (Fig. 3). This is however not unique to TLStab, but common to all benchmarks. Similarly, for TLImm, in the out-of-distribution SARS-Cov-2 viral peptides, AUPRC is well above the baseline, but still low (Fig. 5). We hypothesize that this is still due to limited data; while transfer learning can provide a substantial boost in performance in the presence of small datasets, to further boost overall performance, more training data is needed. Additionally, when TLImm was tested on the TESLA neoepitope dataset [24], its performance was still better when compared to other epitope identification tools, but still lower than some BA/EL predictors. As such, usage of TLImm on neoepitope identification, while competitive, is still limited and not recommended. We hypothesize that the reason for this is the innate feature-related differences between viral peptides and neoepitopes as reported in [27]. These differences have been shown to lead to underperforming models in the literature [28]. As such, future work will emphasize on collecting a neoepitope-specific training dataset, and repeat the train/test process that is presented in this work, thus leading to a neoepitope-specific model that exhibits better generalization performance on the task of neoepitope identification.

## Supplementary Material

1

2

3

4

5

6

7

8

9

## Data Availability

The Python code of both TLStab and TLImm is freely available at https://github.com/KavrakiLab/TL-MHC.
