# Supplementary material for: Transfer learning improves pMHC kinetic stability and immunogenicity predictions
Source: Immunoinformatics (Amst). Author manuscript; Available in PMC 2024 Apr 4. (PMC10994007; doi:10.1016/j.immuno.2023.100030)
Supplement: 8 [file NIHMS1977163-supplement-8.pdf]

Supplementary Methods

Extended Methods

Neural Network Architectures

The neural network models that were chosen for BA/EL prediction, as well as for stability/immunogenicity fine-tuning, are inspired by existing work in the literature. Specifically, we follow conventions already proposed by [1, 2], and employ 3-layer Multi-Layer Perceptrons (MLPs). The input to the MLP is the 45-mer aligned form of the peptide, as proposed in [2], concatenated with the 34-amino acid pseudo-sequence of the MHC, as defined in [1]. Each amino acid of the concatenated pMHC sequence input is represented as a 21-dimensional feature vector, with features being the BLOSUM62 substitution matrix [3] row of this particular amino acid (20×20 BLOSUM62 matrix extended to 21×21 in order to contain the neutral amino acid "X"). As such, in total, the input to the MLP is a 1659-dimensional BLOSUM62 feature vector. The output of the neural network is 2-dimensional, since it is trained on both BA (continuous) and EL (binary) data, which are considered as separate tasks [4]. As such, the loss function that was used to train the MLPs is the summation of the Mean Squared Error (MSE) loss, used for BA (continuous) points, plus the Binary Cross Entropy (BCE) loss, used for EL (binary) points:

$$Loss = \begin{cases} \frac{1}{n}\sum(y - \hat{y}), & \text{if BA} \\ -\frac{1}{n}\sum(y \cdot \log \hat{y} + (1-y)\log(1-\hat{y}), & \text{if EL} \end{cases} \tag{1}$$

We trained the weights of MLPs using Stochastic Gradient Descent (SGD). In **Supplementary Table S7**, the reader can find the set of values that the optimal hyperparameters were chosen from during the model selection step. The hyperparameter values that minimized the aforementioned loss were chosen as optimal.

Knowledge Transfer Approaches for Stability and Immunogenicity prediction

Inspired by the different models proposed in the literature that use BA data/predictions, we built the following MLP architectures for a complete benchmarking on the task of pMHC stability and peptide immunogenicity prediction (see Supplementary Figure S10 for an overview of the knowledge transfer methods):

1. Baseline MLP: Here, given a peptide + MHC sequence input, the MLP predicts pMHC stability/immunogenicity values. This architecture does not use any BA/EL information, and it just optimizes the MLP weights solely based on stability/immunogenicity data (Supplementary Figure S10A).

2. Consensus Scoring: For the consensus scoring method, we follow the same process as previously proposed in [5, 6]. NetMHCstab and NetMHCstabpan both use a simple linear model where BA and Stability values are mixed. Equivalently, our final predictions are a weighted average of the trained stability/immunogenicity prediction MLP and a 0 to 1 BA value (Supplementary Figure S10B):

$$pred = \alpha \cdot Stab + (1-\alpha) \cdot BA \tag{2}$$

   The weight α is a hyperparameter that is optimized during the 10-fold CV. Instead of finding an α value per fold, we calculated the α value that gives the best performance when considering all folds.

3. BA as a feature: This MLP architecture is similar to the baseline, with the only difference being that BA predictions from NetMHCpan4.1 [7] are being used as features for stability/immunogenicity prediction (Supplementary Figure S10C). This is inspired by PRIME [8, 9], although PRIME is not using the whole pMHC sequence, but frequencies of the peptide amino acids that exert minimal impact on pMHC BA. Still, the effect that the BA feature will have during training will be similar, if not equivalent.

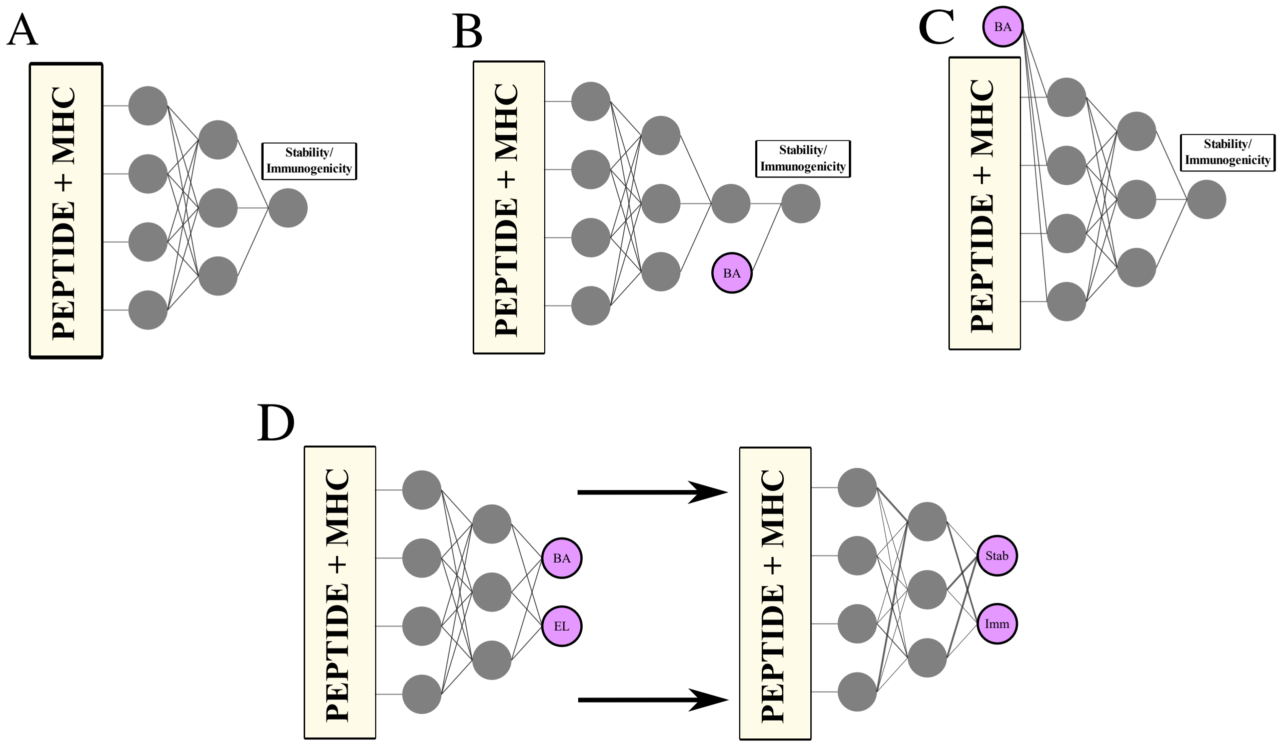

Supplementary Figure S10: Different architectures that use BA predictions at different degrees for pMHC stability and peptide immunogenicity predictions. **(A)** A baseline predictor that is not using affinity. **(B)** A baseline predictor, whose predictions are linearly mixed with affinities. **(C)** A baseline predictor with the additional BA prediction feature. **(D)** TLStab & TLImm: A BA/EL predictor similar to NetMHCpan4.1 is finetuned to stability/immunogenicity tasks. This is achieved by refining the MLP weights through task-specific training.

4. Fine-tuning: The fine-tuning method, which we used to train TLStab and TLImm, is equivalent to the baseline MLP. However, the MLP weights were loaded from our pre-trained BA predictor, instead of them being randomly initialized. The BA output of the network was then re-purposed for pMHC stability prediction, and the EL output of the network was re-purposed for immunogenicity prediction (Supplementary Figure S10D and **Figure 1**). It is worth noting that, during the training process, all weights of the network are subject to change, and no weights/layers of weights are frozen.

5. Frozen layers: A variant of the fine-tuning method (Supplementary Figure S10D), here the same training process is followed, but all layers except the penultimate layer (the last before the neural network output layer) are frozen and are not being fine-tuned to the downstream task.

6. New layer: Also a variant of the fine-tuning method (Supplementary Figure S10D), here the last layer is replaced with a new, task-specific layer, with randomly initialized weights.

TLStab

Training for pMHC kinetic stability prediction

For both training and test pMHC stability sets, we used a transformation from hours to 0-1 values, as reported in [5, 6]: $s = 2^{-\frac{t_0}{t_H}}$, s here being the 0-1 value, t_H the actual half-life measurement, and t_0 an allele-related threshold. Contrary to [5], since we are building pan-allele stability predictors, we did not use different per-allele thresholds t_0 in the transformation formula, but we rather used the value of 1 for

all alleles, the same way it is proposed in [6]. As pMHC stability is transformed to a 0-1 continuous value, for all aforementioned architectures (Supplementary Figure S10), we used the MSE loss for training. The SGD optimizer was used for optimizing/fine-tuning the weights. Model selection was performed by choosing the set of hyperparameters that minimize the loss from a pool of values (**Supplementary Table S7**). We found that learning rates between 0.0001 and 0.00001 achieved better performance on the 10-fold CV. These values constitute a good middle ground between altering the neural network weights significantly (a result of a higher learning rate $> 10^{-3}$) and altering the neural network weights very slightly at each epoch, which can result in underfitting/getting stuck in local minima (a result of a lower learning rate $< 10^{-6}$)

Ebola and Pox virus datasets

Both the Ebola and Pox virus peptide datasets were downloaded from their respective source in IEDB [10, 11] (downloaded on October 2022). The Ebola virus dataset contains 1901 unique peptide-HLA pairs, while the Pox virus dataset contains 931 unique peptide-HLA pairs. As reported in the description of the datasets, measurements were taken through the *Beckman Coulter iTopia Epitope Discovery System* [12]. The iTopia assay comprises of three main assays: A) An Affinity Assay, where the relative BA of each peptide-HLA pair is characterized and expressed in terms of an ED50 value, B) An Off-rate Assay, where the relative rate of dissociation of each peptide-HLA pair is characterized and expressed in terms of an half-life value measured in hours (h), and C) A peptide-binding assay, which characterizes whether a peptide-HLA is a binder or a non-binder through a score referenced as *iScore*, valued from 0-100%. As such, for both datasets and for each peptide-HLA, there exist 3 different values corresponding to each of the assays. Peptide-HLA pairs receiving a *iScore* of $\leq 30\%$ are considered non-binders [12]. As such, we filtered any non-binders based on the *iScore* value. This results in 1023 peptide-HLA pairs for the Ebola virus dataset, and 541 peptide-HLA pairs for the Pox virus dataset, each with an ED50 value and an half-life value measured in h.

TLImm

Training for peptide Imunogenicity prediction

In the case of immunogenic/non-immunogenic labels, we trained all networks using BCE loss, while in the case of the continuous immunogenicity strength value, we used the MSE loss. Again, the hyperparameters that lead to minimum loss were chosen in the model selection step (**Supplementary Table S7**). All networks were trained/fine-tuned through SGD. As with the peptide stability experiments, we also found here that learning rates between 0.0001 and 0.00001 achieved better performance on the 5-fold CV.

Filtering/Labeling the Immunogenicity Training Dataset

Dataset Labeling: Most of the existing approaches treat the peptide immunogenicity prediction problem as a binary classification task, where the positive label translates to an peptide being immunogenic and the negative label being the non-immunogenic instance. DeepImmuno employed a beta-binomial model instead to assign a continuous immunogenicity strength score for each data point, taking into account not just the existence of an immunogenic response, but also the frequency and strength of response in different subjects [13]. We experimented with both labeling schemes in the same training dataset.

Dataset Balancing: Inspired by the immunogenicity model evaluation study by [14], we sought to balance our training set by filtering out data points, with the purpose of equalizing the immunogenic/non-immunogenic balance ratio between different alleles. We therefore sub-sampled the dataset, in order to have a stardard per-allele immunogenic ratio. The optimal ratio was assessed by a) the resulting number of data points after the subsampling, and b) the number of total positive (immunogenic) instances in the filtered dataset. While there are smaller ratios ($< 3\%$) that end up in a slightly higher number of data points, there are very few positive instances to learn from as a result, so we opted for a 60% immunogenic ratio, which forms a very convenient local maximum (Supplementary Figure S11). The total number of data points for this filtered set amounts to 4812 instances in total.

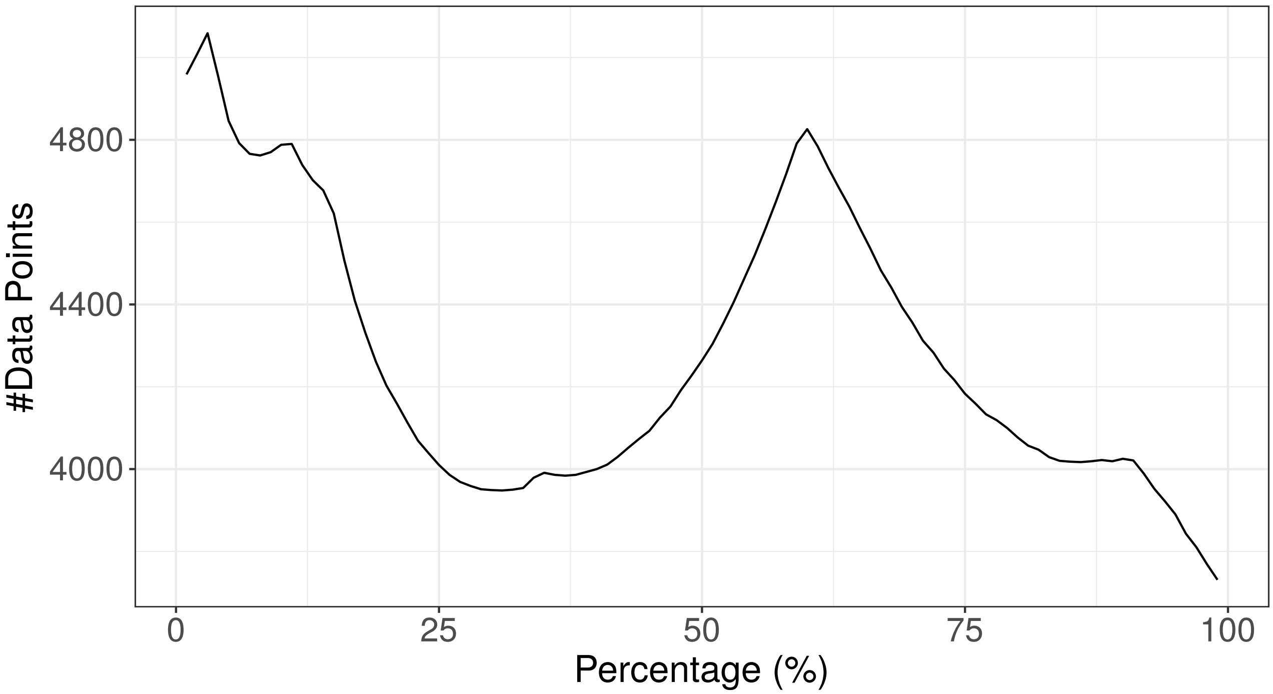

Supplementary Figure S11: Number of datapoints for different per-allele immunogenic label ratios. Based on the curve, the ratio that also keeps the largest number of points, while also resulting in the adequate number of positive instances to learn from, is around 60% immunogenic peptides per allele.

It is important to underline here that, through this subsampling process, there will be a substantial amount of datapoints that won't be considered in the training process. To this end, we repeat the subsampling process three different times, creating three different datasets as a result. Repeating 5-fold nested CV for each dataset thus results in TLImm essentially being an ensemble of fifteen different MLPs.

Combining all variants discussed above, we created four different configurations of the training dataset:

1. An unbalanced training dataset with immunogenic/non-immunogenic binary labels. This is similar to most immunogenicity prediction approaches in the literature [14].
2. An unbalanced training dataset with continuous immunogenic strength labels, similar to [13].
3. A 60%/40% balanced per-allele training dataset with immunogenic/non-immunogenic binary labels.
4. A 60%/40% balanced per-allele training dataset with continuous immunogenic strength labels.

This was done with the purpose of examining which configuration leads to the best model, as being assessed on benchmark test datasets.

Training a simple Random Forest for explanatory analysis

To understand and examine in greater detail the effect of having an per-allele imbalanced vs. a per-allele balanced training dataset during training, we trained a simple Random Forest (RF) peptide immunogenicity model. The RF was trained on the same features that we trained the MLP architectures on. To avoid performing the peptide alignment process that could obscure explainability, we only used the 9-mer peptides of the dataset to train the RF. This way, imputation with neutral amino acid 'X' that was employed to account for peptides of different length was not necessary. The input to the RF was the 9-mer peptide sequence + the MHC pseudo-sequence To assess the feature importance for each amino-acid in the input

(either from the peptide or the MHC), we perform a mean averaging of the feature importances of the 21-dimensional vector representing each amino acid. The R package *ranger* was used for building and training the RF [15]. The number of trees was set to 500, and all other parameters were set to their default values.

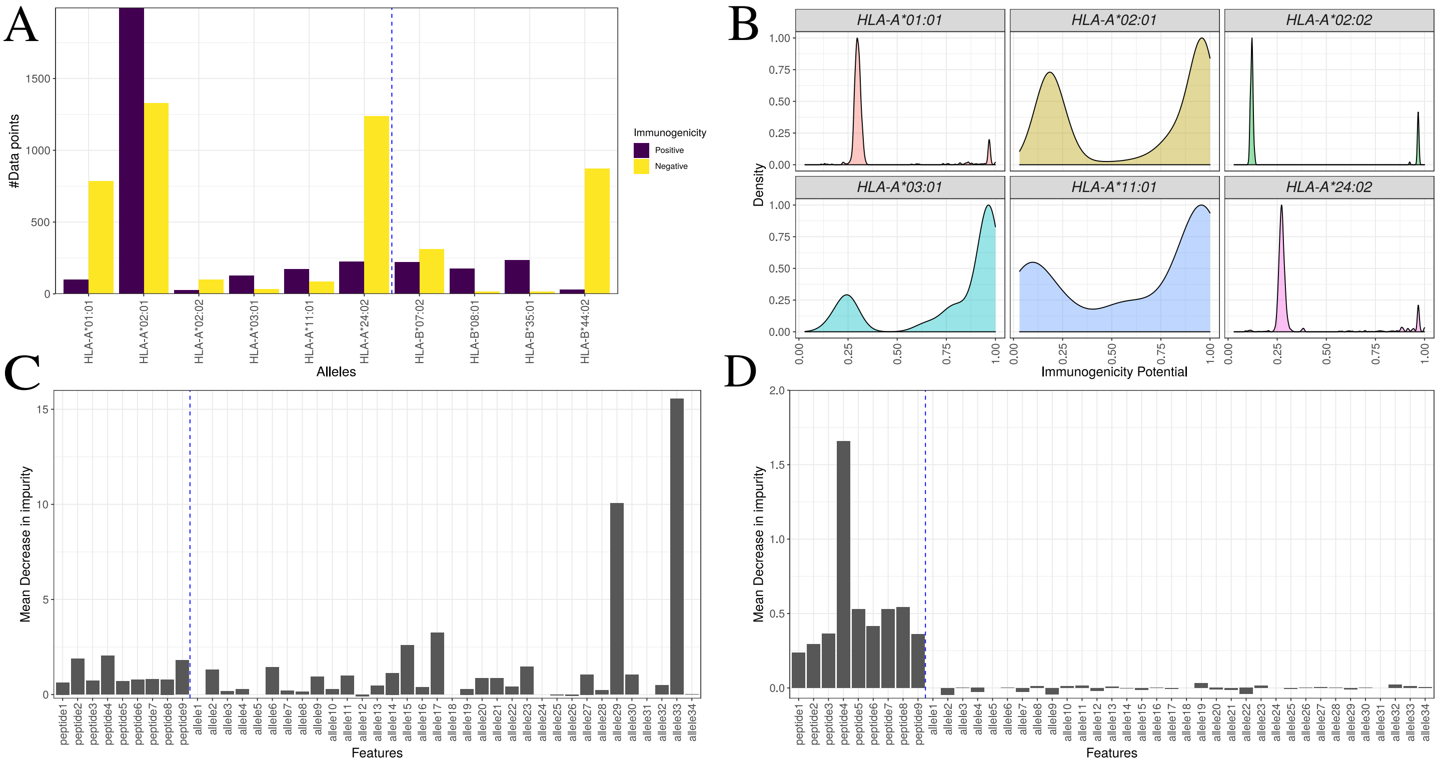

Supplementary Figure S12: (A) Distribution of immunogenic/non-immunogenic labels on the 10 most prominent alleles of the imbalanced training dataset. (B) Distribution of immunogenicity strength labels in selected HLA-A alleles from the imbalanced training dataset. (C) Feature Importances of the RF model when trained on an dataset that is per-allele imbalanced. (D) Feature Importances of the RF model when the dataset is balanced.

Extended Results

TLImm: An ablation study

The study by [14] has previously hinted at the immunogenic label ratio imbalance in benchmark immunogenicity datasets, and the ways that this affects performance on peptide immunogenicity prediction models. Simply visualizing the label distribution of the most prevalent alleles on the IEDB dataset that we used to train TLImm indeed confirms the imbalance (Supplementary Figure S12A). Going further to associate per-allele imbalance with potential faulty models, we examined the features that a baseline immunogenicity model pays attention to the most in an imbalanced dataset. To this end, we trained a simplified Random Forest (RF) model on the same data we trained TLImm (see previous section on training a simple Random Forest for explanatory analysis). The feature importances of the RF model can be seen in Supplementary Figure S12C. A substantial proportion of the feature importance was allocated to the MHC pseudo-sequence related amino acids, which are not amino acids that drive the immune response. We hypothesize that a model trained on an imbalanced dataset focuses on the allele identity features, instead of the peptide amino acids. We subsequently performed a per-allele dataset balancing. When training the RF on the balanced dataset, we observed a very different distribution of feature importances in the peptide amino acid regions (Supplementary Figure S12D). Especially, the peptide residue at position 4 seem to be the most important

for the model to distinguish between an immunogenic vs. non-immunogenic peptide, confirming reported results [13, 16].

Most studies treat the peptide immunogenicity prediction as a binary classification task. The DeepImmuno study experimented with assigning an immunogenicity strength score, based on the number of positive responses in donors and the total number of donors for each pMHC pair [13]. We further experimented with the notion of an immunogenic score as previously defined [13], and tested the effects of training TLImm on a regression task instead of a binary classification task. It is worth clarifying that a mere re-labelling does not solve the per-allele balance issue mentioned above, as the per-allele distributions of different immunogenic scores still differ (Supplementary Figure S12B). Consequently, per-allele balancing is needed even in the continuous case in order to not misguide the trained model.

According to the aforementioned analyses, four different versions of TLImm were created (see Filtering/Labeling section above for more details):

1. TLImm, trained on an imbalanced dataset with binary labels.
2. TLImm + Bal., trained on an balanced dataset with binary labels.
3. TLImm + Cont., trained on an imbalanced dataset with continuous immunogenic strength labels.
4. TLImm + Bal. + Cont., trained on an balanced dataset with continuous immunogenic strength labels.

References

[1] M. Nielsen, C. Lundegaard, T. Blicher, K. Lamberth, M. Harndahl, S. Justesen, G. Røder, B. Peters, A. Sette, O. Lund, S. Buus, Netmhcpan, a method for quantitative predictions of peptide binding to any hla-a and -b locus protein of known sequence, PLOS ONE 2 (2007) 1–10.

[2] T. J. O'Donnell, A. Rubinsteyn, U. Laserson, Mhcflurry 2.0: Improved pan-allele prediction of mhc class i-presented peptides by incorporating antigen processing, Cell Systems 11 (2020) 42–48.e7.

[3] S. Henikoff, J. G. Henikoff, Amino acid substitution matrices from protein blocks., Proceedings of the National Academy of Sciences 89 (1992) 10915–10919.

[4] V. Jurtz, S. Paul, M. Andreatta, P. Marcatili, B. Peters, M. Nielsen, Netmhcpan-4.0: Improved peptide–mhc class i interaction predictions integrating eluted ligand and peptide binding affinity data, The Journal of Immunology 199 (2017) 3360–3368.

[5] K. W. Jørgensen, M. Rasmussen, S. Buus, M. Nielsen, Netmhcstab - predicting stability of peptide-mhc-i complexes; impacts for cytotoxic t lymphocyte epitope discovery, Immunology 141 (2014) 18–26.

[6] M. Rasmussen, E. Fenoy, M. Harndahl, A. B. Kristensen, I. K. Nielsen, M. Nielsen, S. Buus, Pan-specific prediction of peptide–mhc class i complex stability, a correlate of t cell immunogenicity, The Journal of Immunology 197 (2016) 1517–1524.

[7] B. Reynisson, B. Alvarez, S. Paul, B. Peters, M. Nielsen, NetMHCpan-4.1 and NetMHCIIpan-4.0: improved predictions of MHC antigen presentation by concurrent motif deconvolution and integration of MS MHC eluted ligand data, Nucleic Acids Research 48 (2020) W449–W454.

[8] J. Schmidt, A. R. Smith, M. Magnin, J. Racle, J. R. Devlin, S. Bobisse, J. Cesbron, V. Bonnet, S. J. Carmona, F. Huber, G. Ciriello, D. E. Speiser, M. Bassani-Sternberg, G. Coukos, B. M. Baker, A. Harari, D. Gfeller, Prediction of neo-epitope immunogenicity reveals tcr recognition determinants and provides insight into immunoediting, Cell Reports Medicine 2 (2021) 100194.

[9] D. Gfeller, J. Schmidt, G. Croce, P. Guillaume, S. Bobisse, R. Genolet, L. Queiroz, J. Cesbron, J. Racle, A. Harari, Predictions of immunogenicity reveal potent SARS-CoV-2 CD8+ t-cell epitopes, 2022. doi:10.1101/2022.05.23.492800.

[10] K. J. Weinhold, G. D. Tomaras, Y. Cai, K. Plonk, P. Scott, S. K. Nair, Identification of cd8+ t cell epitopes in ebola virus, https://www.iedb.org/reference/1014192, 2009.

[11] K. J. Weinhold, G. D. Tomaras, Y. Cai, K. Plonk, P. Scott, S. K. Nair, Identification of cd8+ t cell epitopes for poxvirus, https://www.iedb.org/reference/1014194, 2009.

[12] M. Wulf, P. Hoehn, P. Trinder, Identification of human MHC class i binding peptides using the iTOPIA™- epitope discovery system, in: Epitope Mapping Protocols, Humana Press, 2009, pp. 361–367. doi:10.1007/978-1-59745-450-6_26.

[13] G. Li, B. Iyer, V. B. S. Prasath, Y. Ni, N. Salomonis, DeepImmuno: deep learning-empowered prediction and generation of immunogenic peptides for T-cell immunity, Briefings in Bioinformatics 22 (2021). Bbab160.

[14] P. R. Buckley, C. H. Lee, R. Ma, I. Woodhouse, J. Woo, V. O. Tsvetkov, D. S. Shcherbinin, A. Antanaviciute, M. Shughay, M. Rei, A. Simmons, H. Koohy, Evaluating performance of existing computational models in predicting CD8+ T cell pathogenic epitopes and cancer neoantigens, Briefings in Bioinformatics 23 (2022). Bbac141.

[15] M. N. Wright, A. Ziegler, ranger: A fast implementation of random forests for high dimensional data in C++ and R, Journal of Statistical Software 77 (2017) 1–17.

[16] J. J. A. Calis, M. Maybeno, J. A. Greenbaum, D. Weiskopf, A. D. D. Silva, A. Sette, C. Keşmir, B. Peters, Properties of MHC class i presented peptides that enhance immunogenicity, PLoS Computational Biology 9 (2013) e1003266.
