# Supplementary material for: Transfer learning improves pMHC kinetic stability and immunogenicity predictions
Source: Immunoinformatics (Amst). Author manuscript; Available in PMC 2024 Apr 4. (PMC10994007; doi:10.1016/j.immuno.2023.100030)
Supplement: 9 [file NIHMS1977163-supplement-9.zip › Supplementary_Figure_11.pdf]

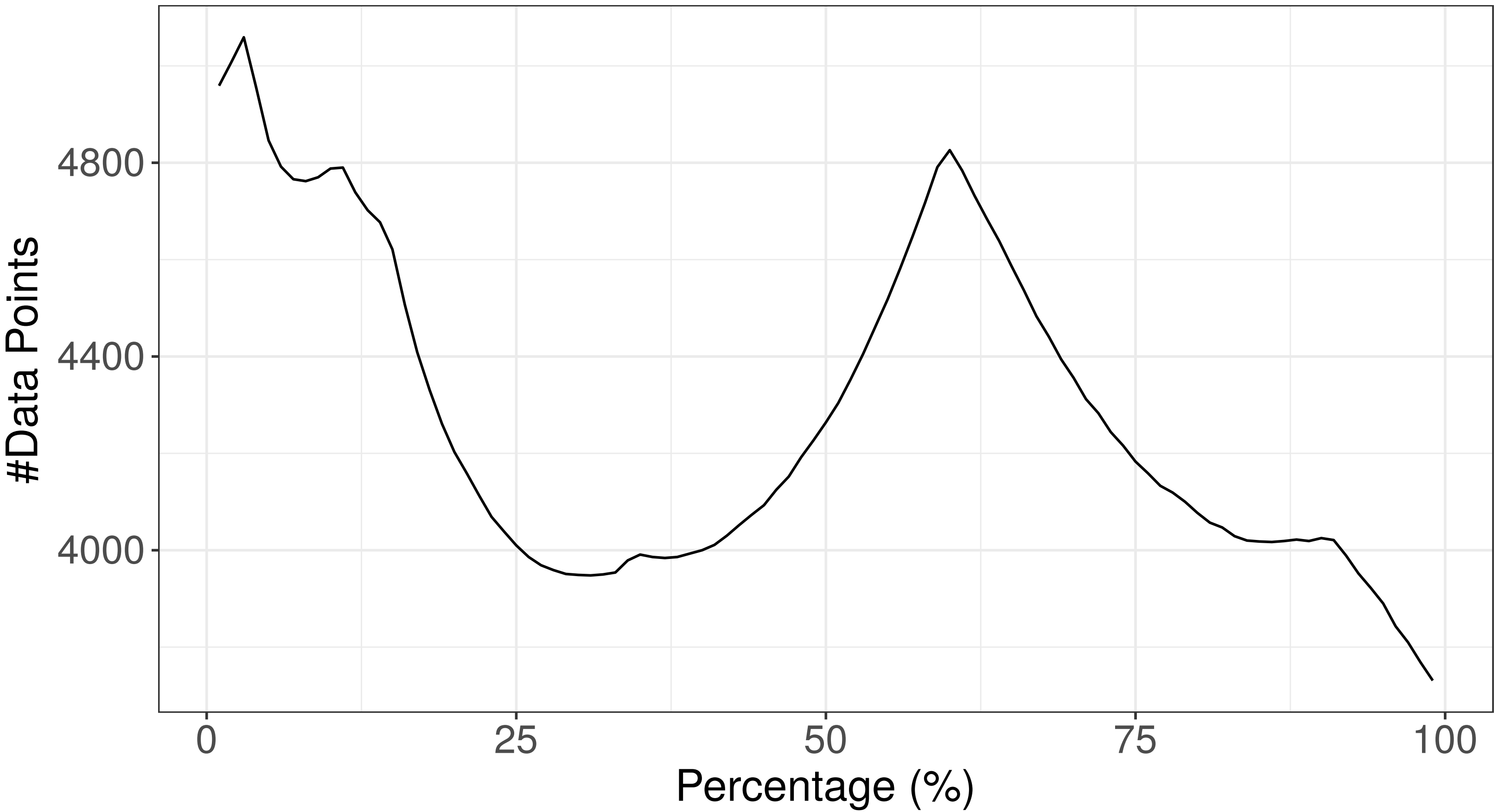

Supplementary Figure S11: Number of datapoints for different per-allele immunogenic label ratios. Based on the curve, the ratio that also keeps the largest number of points, while also resulting in the adequate number of positive instances to learn from, is around 60% immunogenic peptides per allele.
