# Supplementary material for: Transfer learning improves pMHC kinetic stability and immunogenicity predictions
Source: Immunoinformatics (Amst). Author manuscript; Available in PMC 2024 Apr 4. (PMC10994007; doi:10.1016/j.immuno.2023.100030)
Supplement: 9 [file NIHMS1977163-supplement-9.zip › Supplementary_Figure_12.pdf]

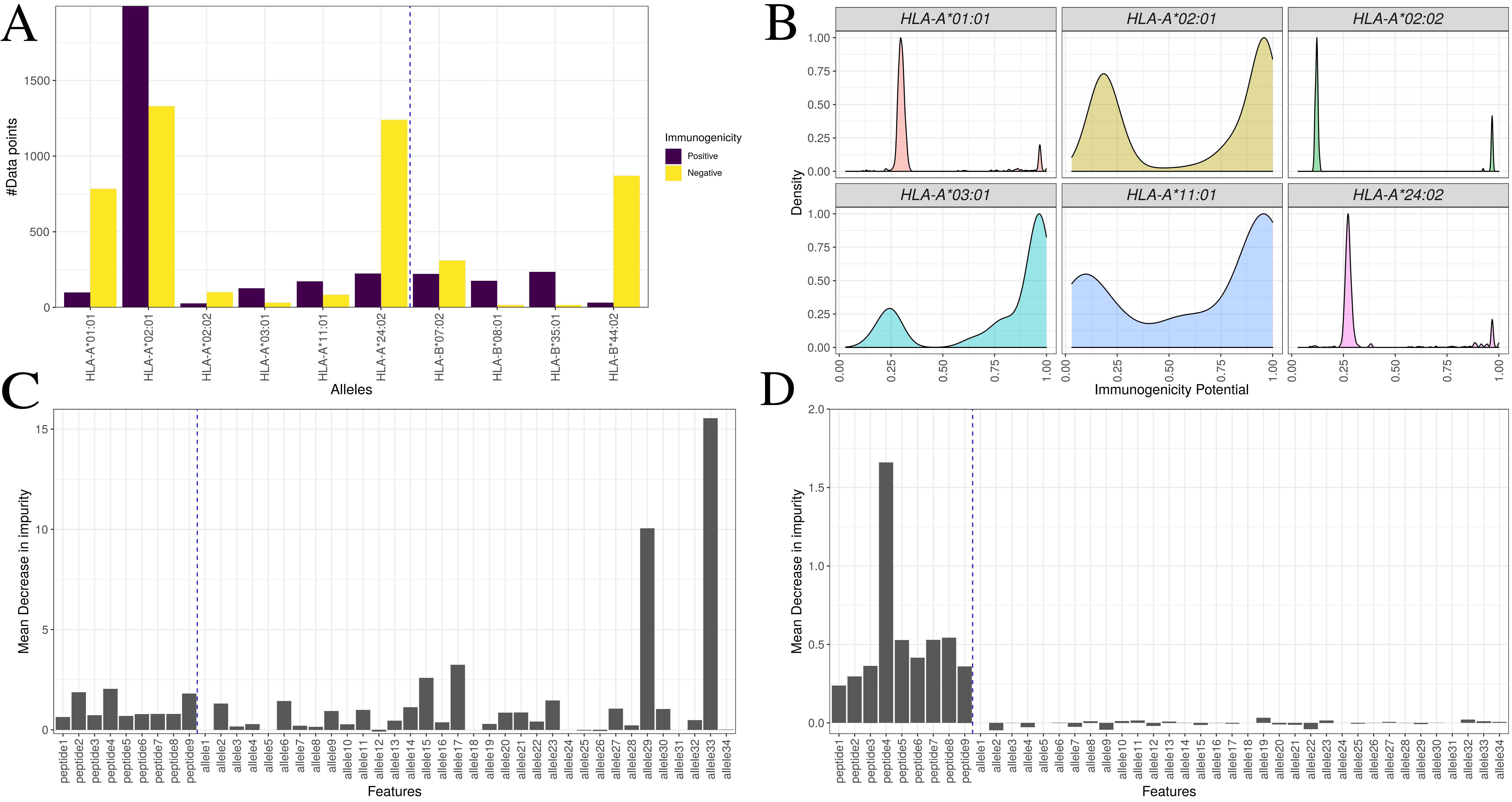

Supplementary Figure S12: **(A)** Distribution of immunogenic/non-immunogenic labels on the 10 most prominent alleles of the imbalanced training dataset. **(B)** Distribution of immunogenicity strength labels in selected HLA-A alleles from the imbalanced training dataset. **(C)** Feature Importances of the RF model when trained on an dataset that is per-allele imbalanced. **(D)** Feature Importances of the RF model when the dataset is balanced.
