# Supplementary material for: Transfer learning improves pMHC kinetic stability and immunogenicity predictions
Source: Immunoinformatics (Amst). Author manuscript; Available in PMC 2024 Apr 4. (PMC10994007; doi:10.1016/j.immuno.2023.100030)
Supplement: 9 [file NIHMS1977163-supplement-9.zip › Supplementary_Figure_1.pdf]

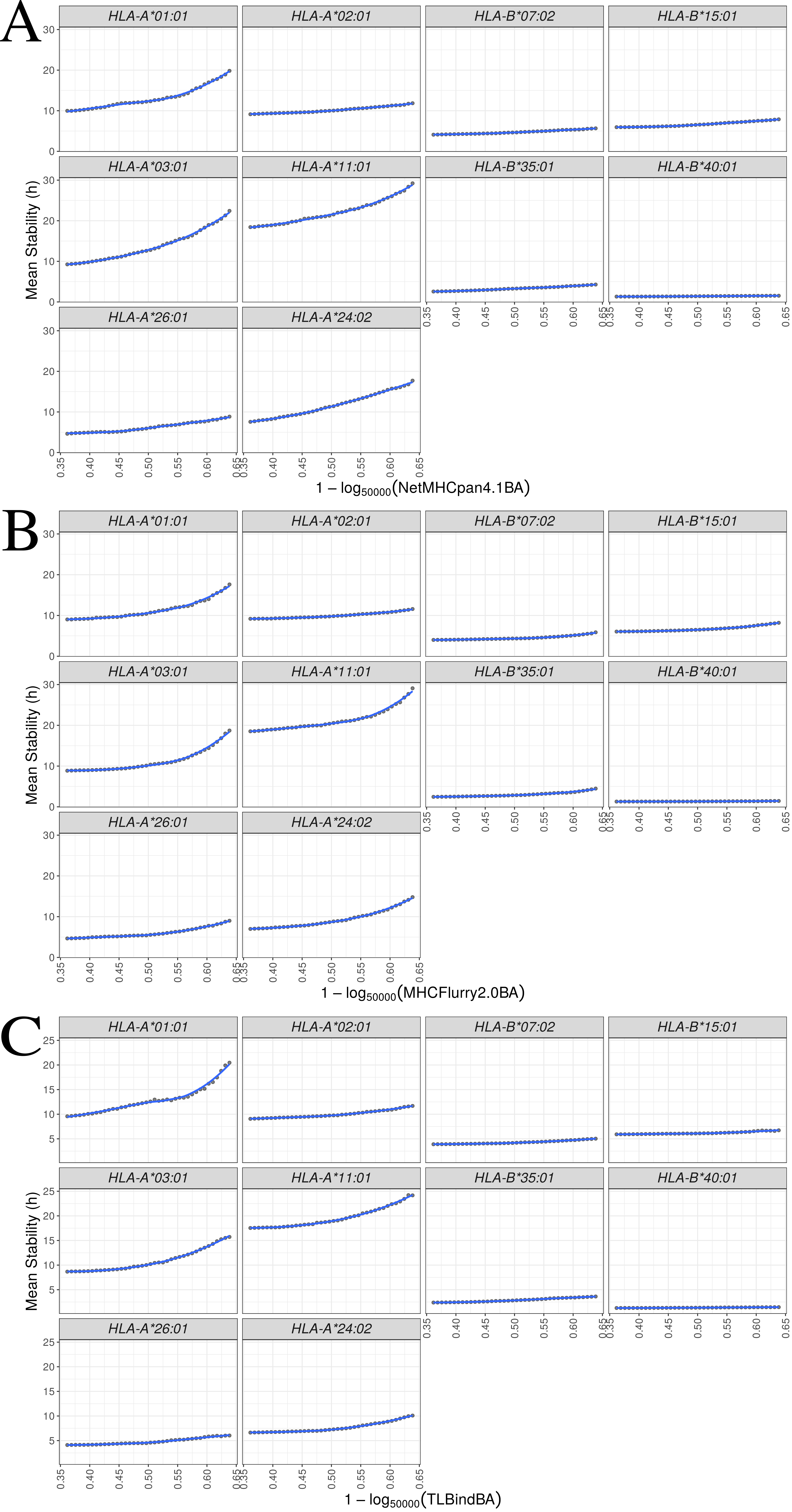

Supplementary Figure S1: **(A)** Relationship between BA predictions from NetMHCpan4.1 and stability values. The y-axis depicts the mean stability values of peptides that have a better predicted BA than the threshold (x-axis). **(B)** Relationship between BA predictions from MHCFlurry2.0 and stability values. The y-axis depicts the mean stability values of peptides that have a better predicted BA than the threshold (x-
