# Supplementary material for: Transfer learning improves pMHC kinetic stability and immunogenicity predictions
Source: Immunoinformatics (Amst). Author manuscript; Available in PMC 2024 Apr 4. (PMC10994007; doi:10.1016/j.immuno.2023.100030)
Supplement: 9 [file NIHMS1977163-supplement-9.zip › Supplementary_Figure_3.pdf]

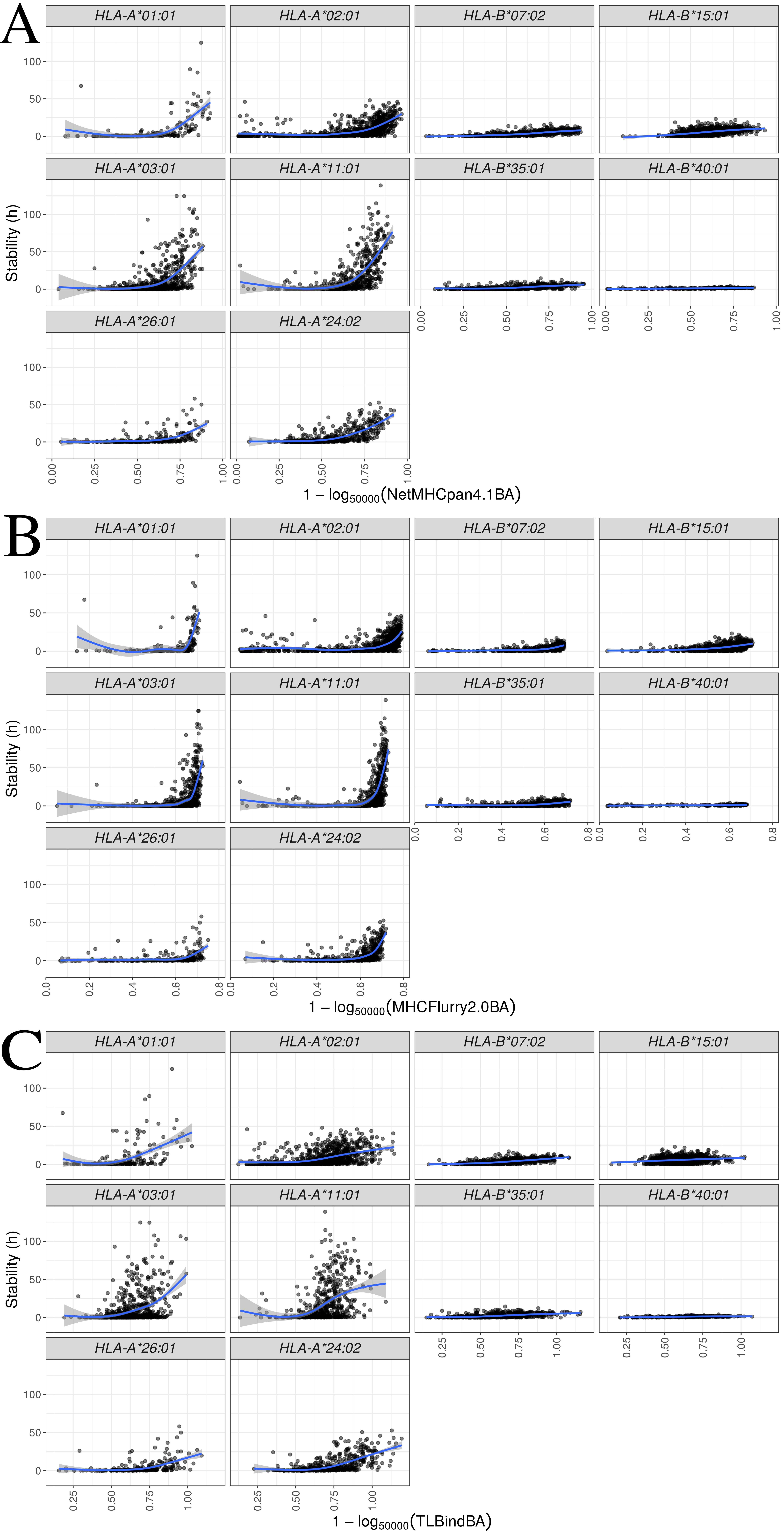

Supplementary Figure S3: **(A)** Relationship between BA predictions and stability values in the NetMHCstab dataset. The y-axis depicts the stability values of peptides (in (h)). The x-axis depicts the scaled NetMHCpan4.1 values of peptides. **(B)** Relationship between BA predictions and stability values in the NetMHCstab dataset. The y-axis depicts the stability values of peptides (in (h)). The x-axis depicts the scaled MHCFlurry2.0 values of peptides. **(C)** Relationship between BA predictions and stability values in the NetMHCstab dataset. The y-axis depicts the stability values of peptides (in (h)). The x-axis depicts the scaled TLBind values of peptides.
