# Supplementary material for: Transfer learning improves pMHC kinetic stability and immunogenicity predictions
Source: Immunoinformatics (Amst). Author manuscript; Available in PMC 2024 Apr 4. (PMC10994007; doi:10.1016/j.immuno.2023.100030)
Supplement: 9 [file NIHMS1977163-supplement-9.zip › Supplementary_Figure_4.pdf]

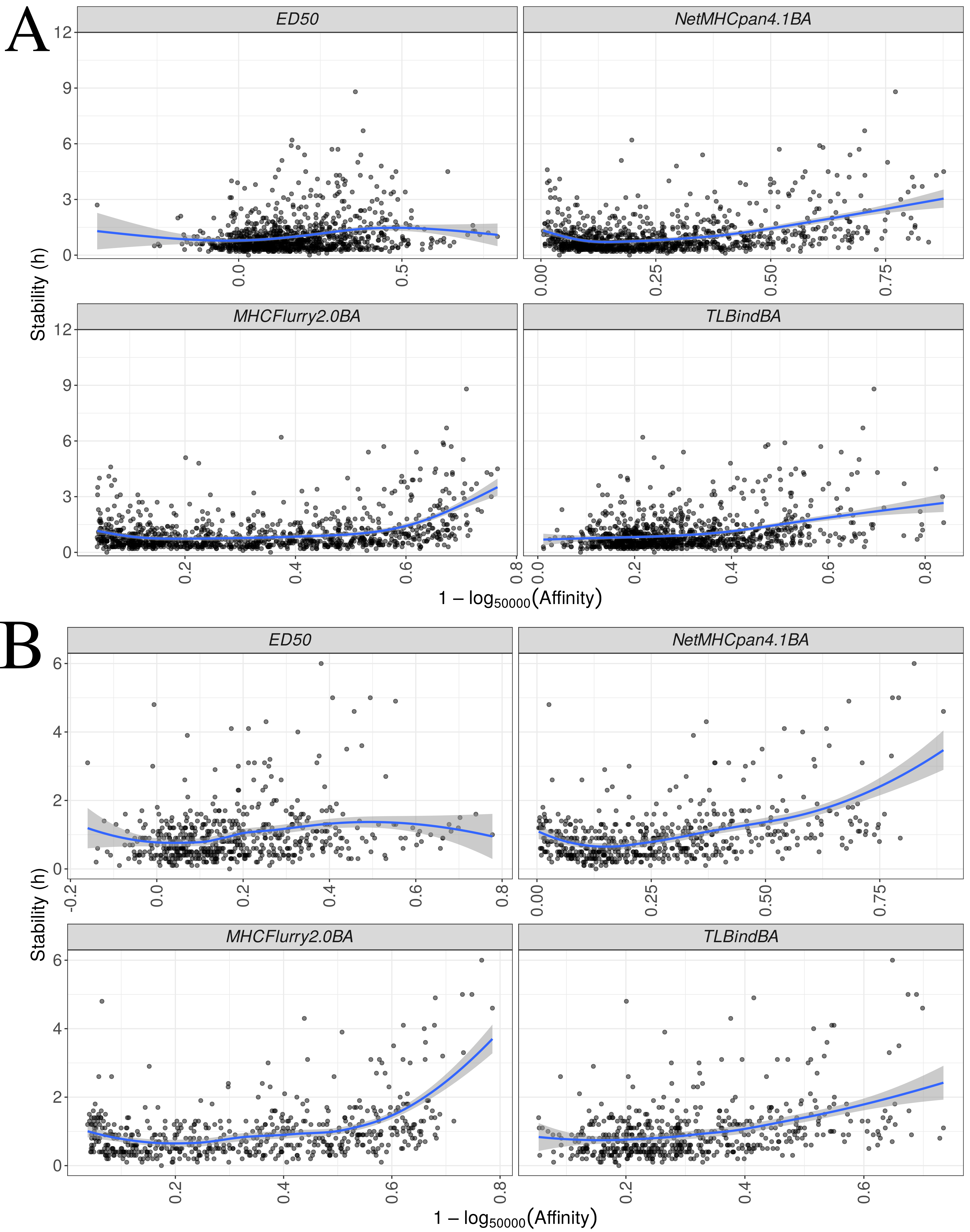

Supplementary Figure S4: (A) Relationship between ED50 values or BA predictions and stability values in the Ebola virus dataset. The y-axis depicts the stability values of peptides (in (h)). The x-axis depicts the scaled ED50/BA prediction values of peptides. **(B)** Relationship between ED50 values or BA predictions and stability values in the Pox virus dataset. The y-axis depicts the stability values of peptides (in (h)). The x-axis depicts the scaled ED50/BA prediction values of peptides.
