# Supplementary material for: Transfer learning improves pMHC kinetic stability and immunogenicity predictions
Source: Immunoinformatics (Amst). Author manuscript; Available in PMC 2024 Apr 4. (PMC10994007; doi:10.1016/j.immuno.2023.100030)
Supplement: 9 [file NIHMS1977163-supplement-9.zip › Supplementary_Figure_5.pdf]

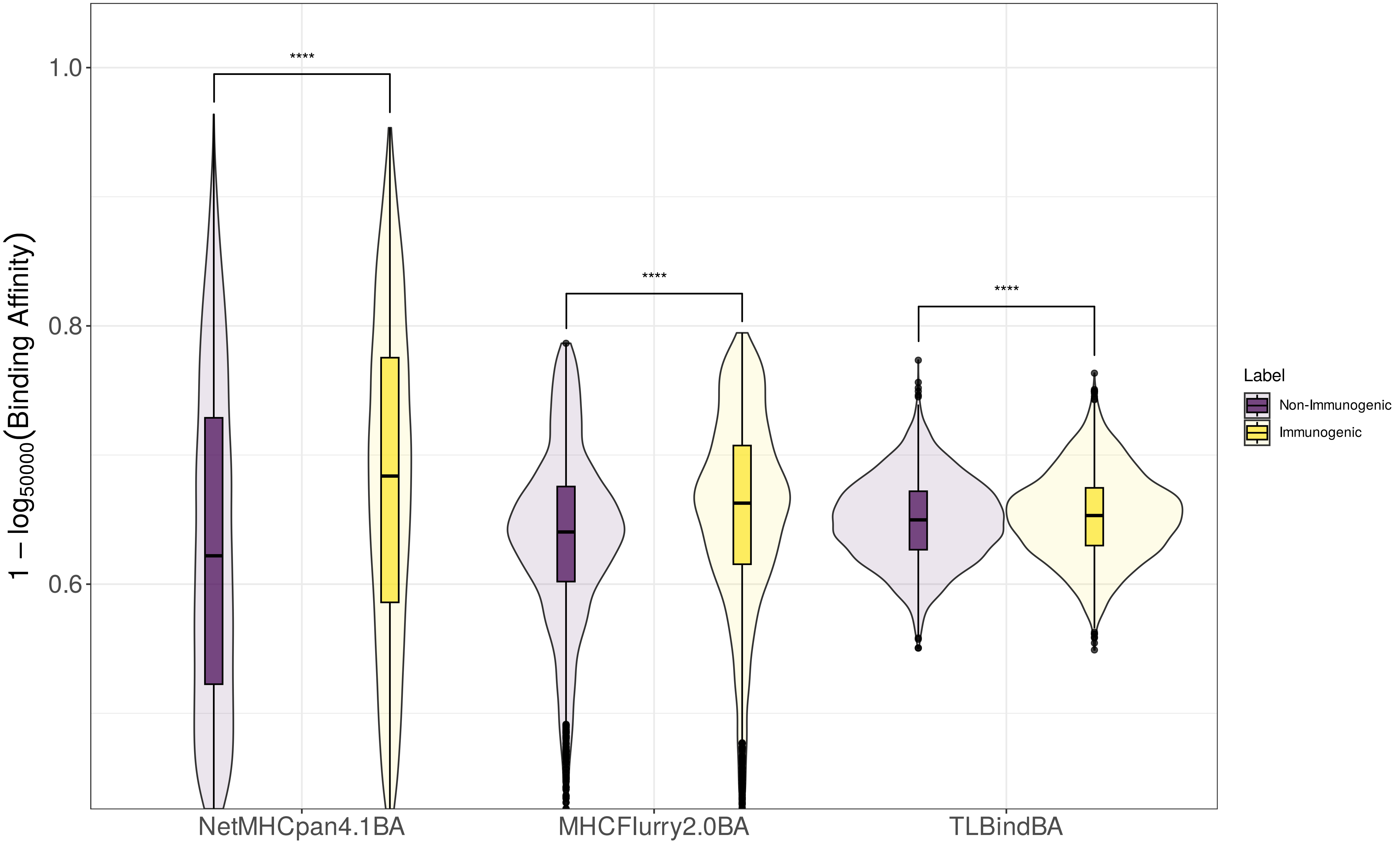

Supplementary Figure S5: Relationship between BA predictions and immunogenic/non-immunogenic labels on IEDB data. NetMHCpan4.1 ($p < 0.0001$), MHCFlurry2.0 ($p < 0.0001$) and TLBind ($p < 0.0001$) affinity predictions on immunogenic peptides are significantly different when compared to non-immunogenic ones.
