# Supplementary material for: Transfer learning improves pMHC kinetic stability and immunogenicity predictions
Source: Immunoinformatics (Amst). Author manuscript; Available in PMC 2024 Apr 4. (PMC10994007; doi:10.1016/j.immuno.2023.100030)
Supplement: 9 [file NIHMS1977163-supplement-9.zip › Supplementary_Figure_6.pdf]

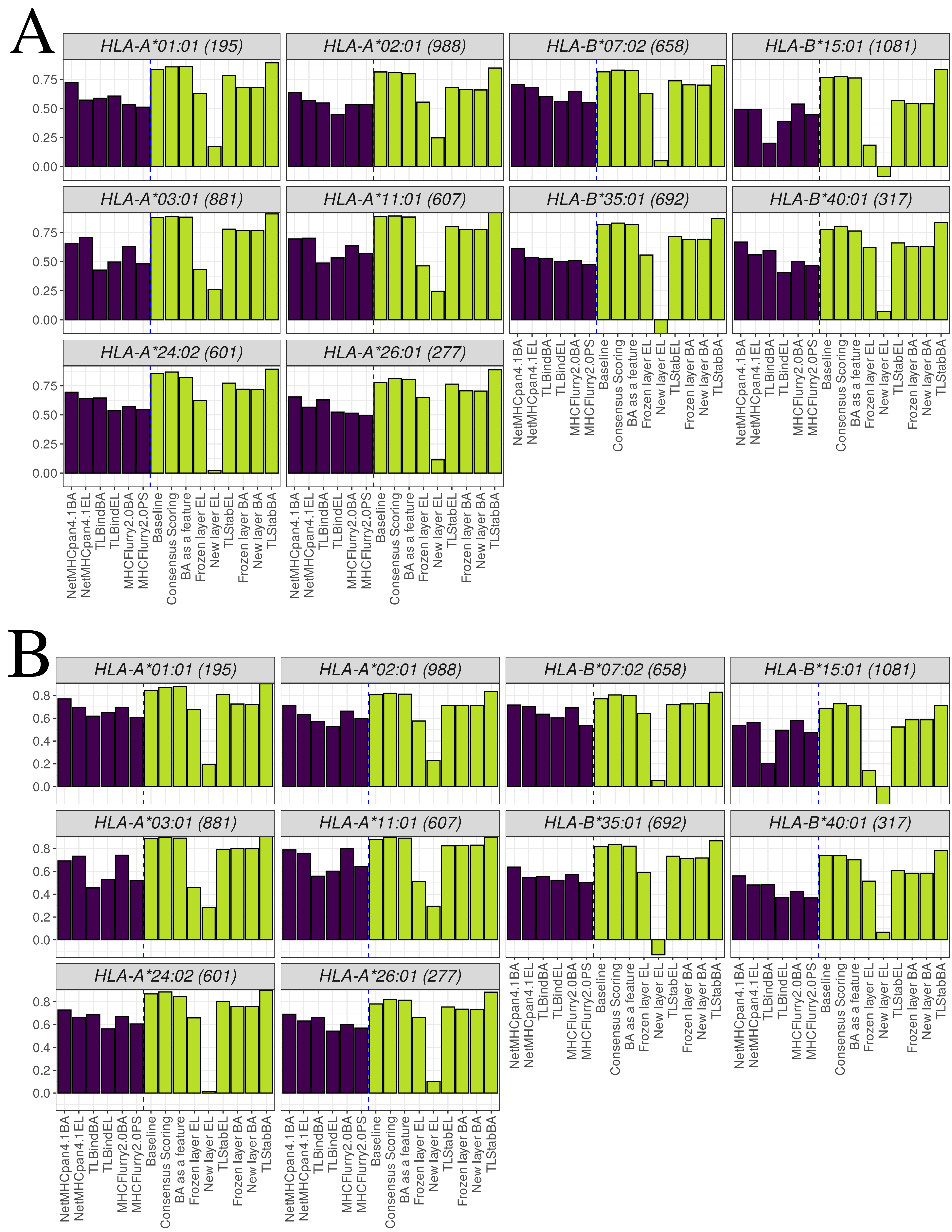

Supplementary Figure S6: Per-allele performance of all benchmarked models on the unbiased 10-fold nested CV experiment. **(A)** Pearson's correlation coefficient. **(B)** Kendall's tau coefficient.
