# Supplementary material for: Transfer learning improves pMHC kinetic stability and immunogenicity predictions
Source: Immunoinformatics (Amst). Author manuscript; Available in PMC 2024 Apr 4. (PMC10994007; doi:10.1016/j.immuno.2023.100030)
Supplement: 9 [file NIHMS1977163-supplement-9.zip › Supplementary_Figure_9.pdf]

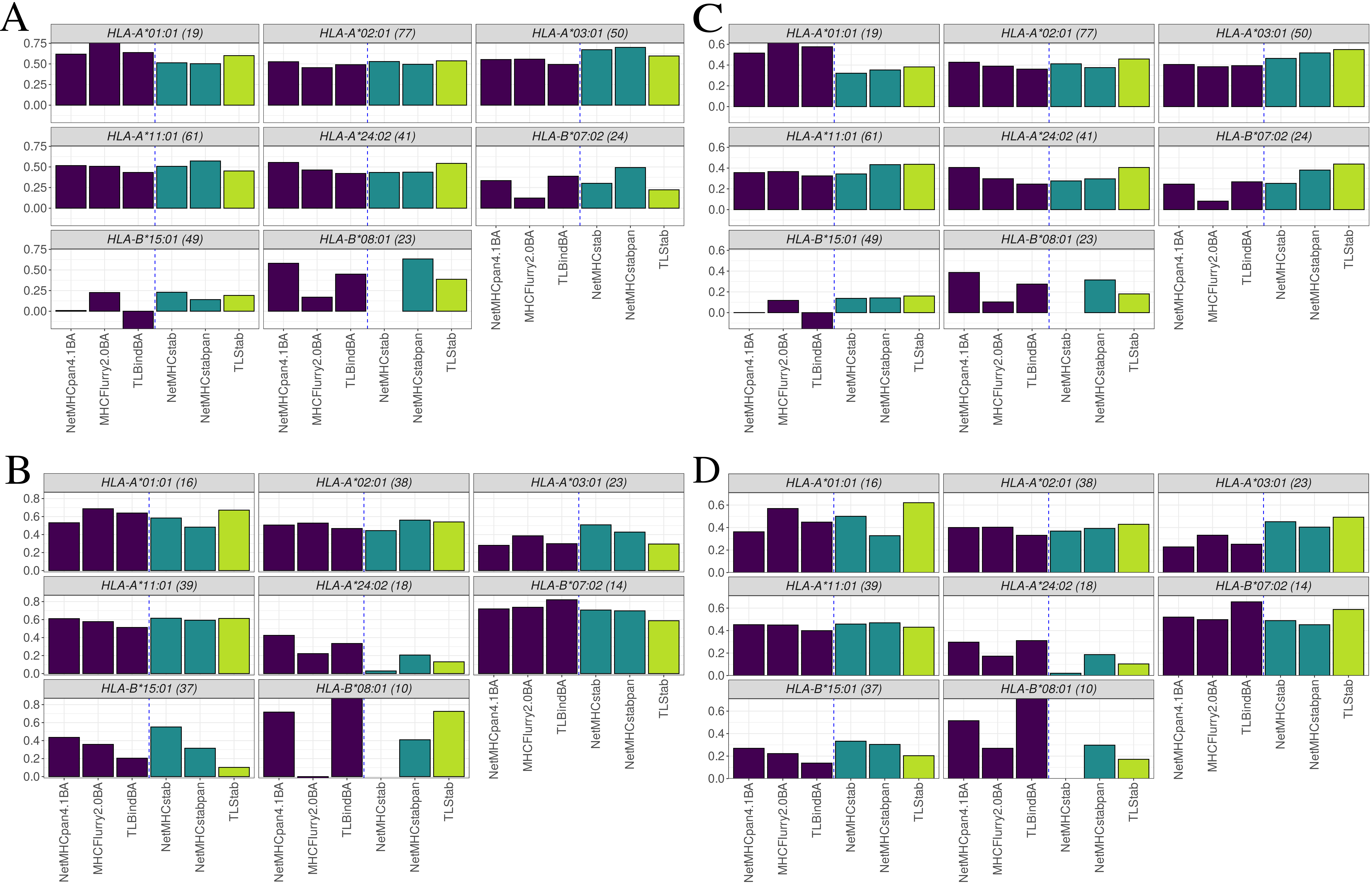

Supplementary Figure S9: **(A)** Pearson's correlation performance of TLStab over other approaches on the filtered (BA predictions < 500 nM) Ebola virus Dataset. On the left part of the blue dashed line, the performance of BA predictors is depticted (blue bars). On the right side, we show the performance of state-of-the-art pMHC stability tools (teal bars) compared to TLStab (dark yellow bar). **(B)** Kendall's tau performance of TLStab over other approaches on the filtered (BA predictions < 500 nM) Ebola virus Dataset. **(C)** Pearson's correlation performance of TLStab over other approaches on the filtered (BA predictions < 500 nM) Pox virus Dataset. **(D)** Kendall's tau performance of TLStab over other approaches on the filtered (BA predictions < 500 nM) Pox virus Dataset.
