# Supplementary figures and images for: Transfer learning improves pMHC kinetic stability and immunogenicity predictions
Source: Immunoinformatics (Amst). Author manuscript; Available in PMC 2024 Apr 4. (PMC10994007; doi:10.1016/j.immuno.2023.100030)

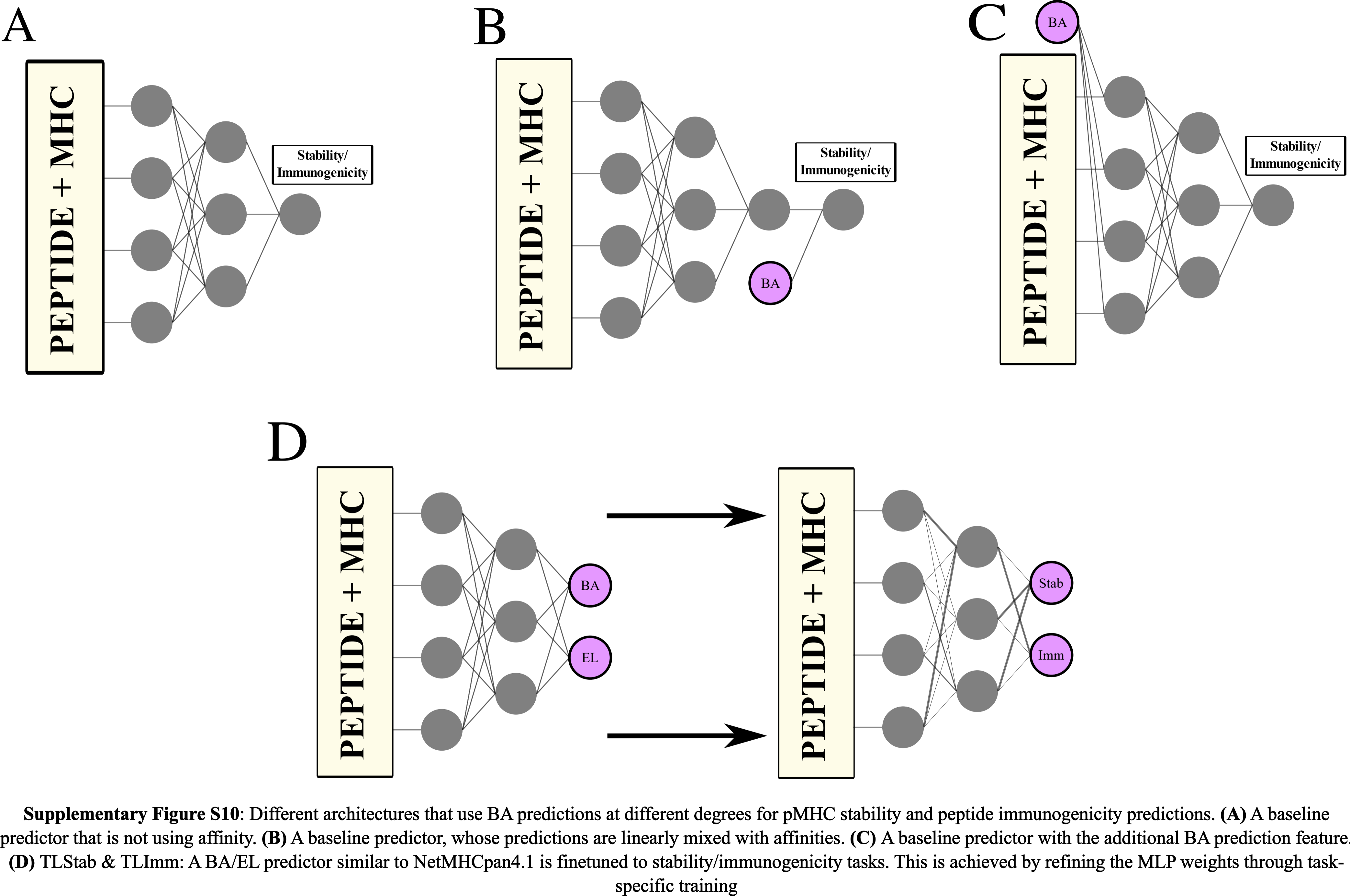

Supplement: 9 [file NIHMS1977163-supplement-9.zip › Supplementary_Figure_10.pdf]

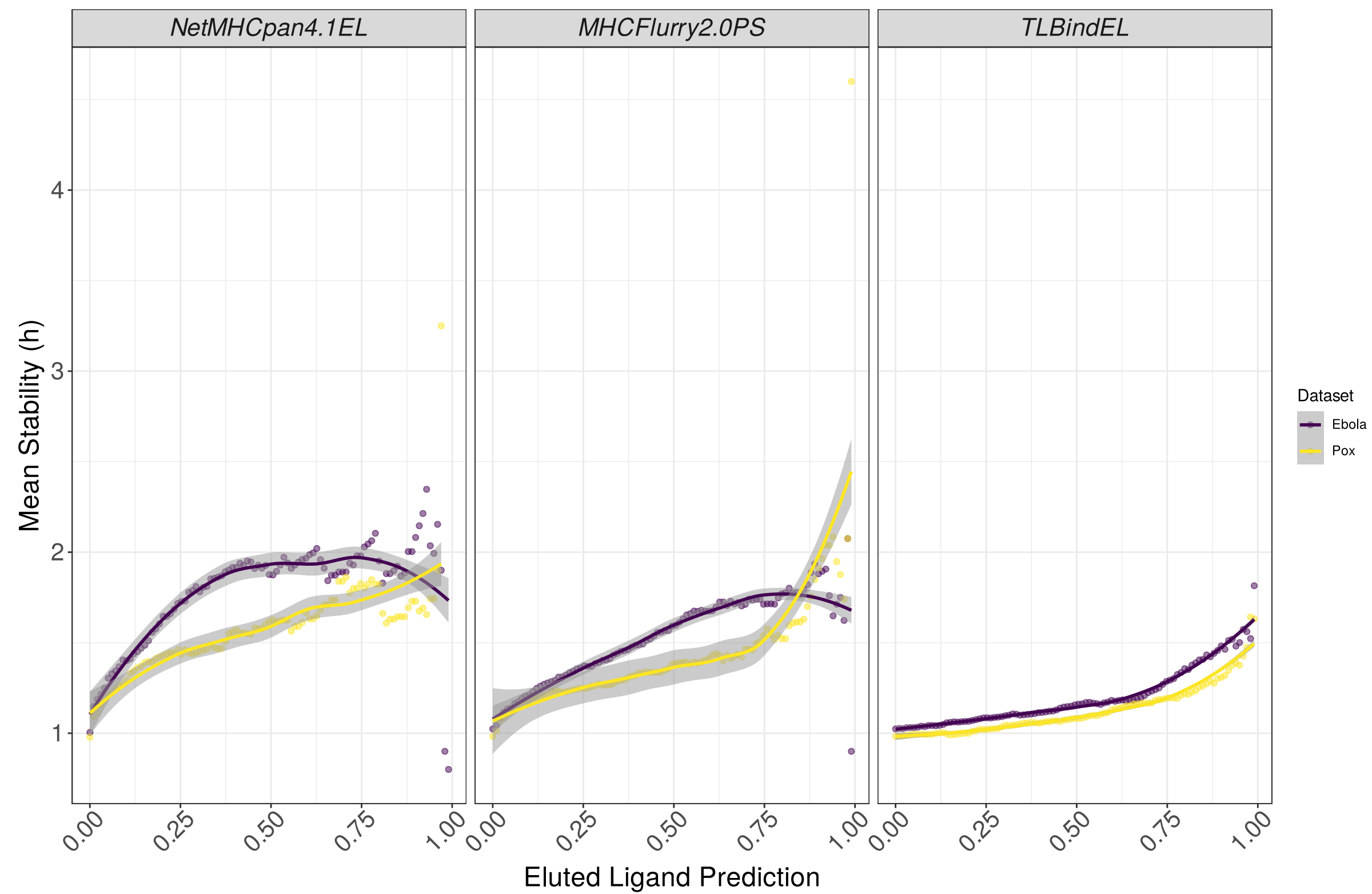

Supplement: 9 [file NIHMS1977163-supplement-9.zip › Supplementary_Figure_2.pdf]
