# Supplementary material for: Transfer learning improves pMHC kinetic stability and immunogenicity predictions
Source: Immunoinformatics (Amst). Author manuscript; Available in PMC 2024 Apr 4. (PMC10994007; doi:10.1016/j.immuno.2023.100030)
Supplement: 9 [file Supplementary_Figure_8.pdf › Supplementary_Figure_8.pdf]

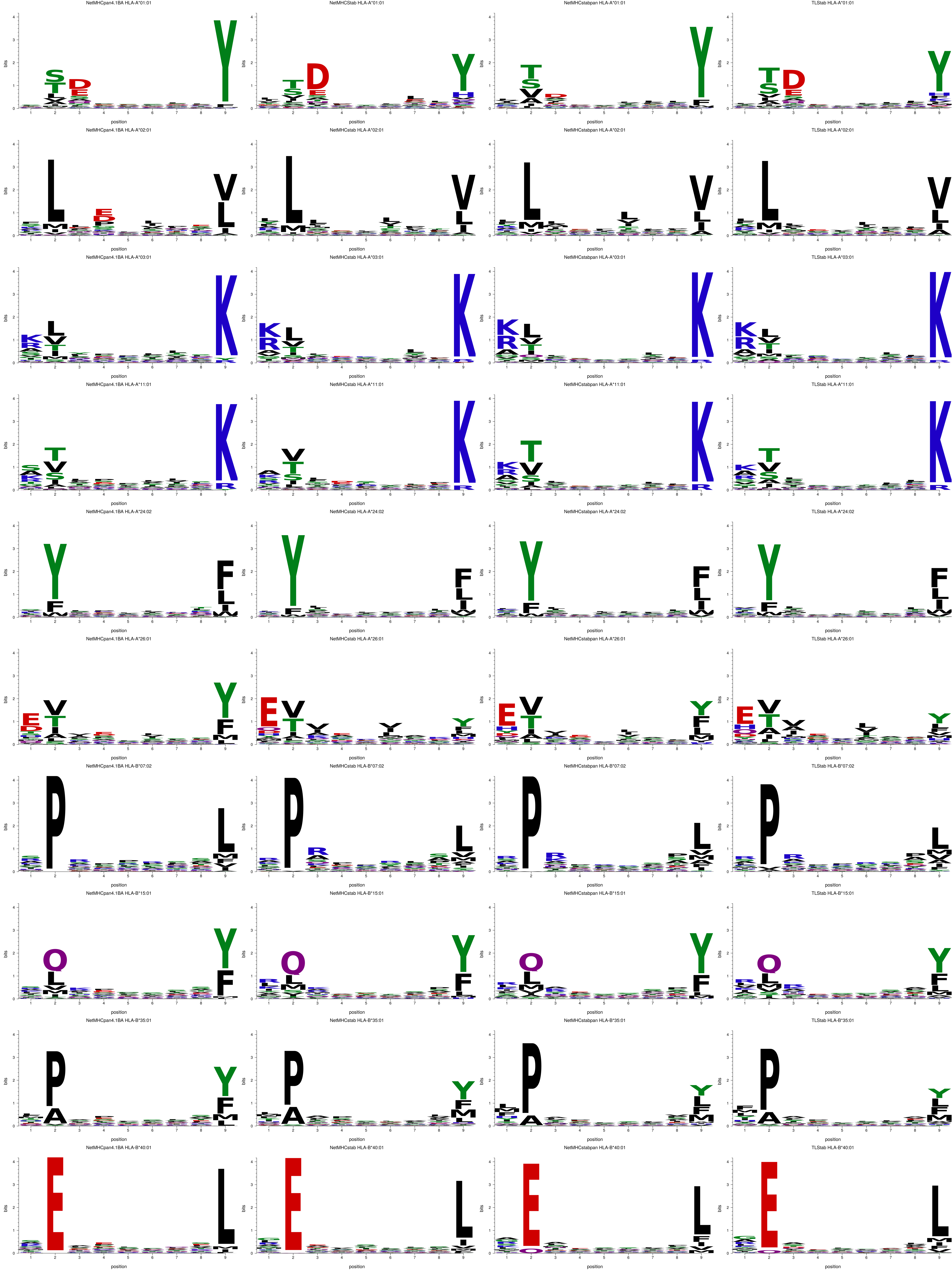
NetMHCpan4.1BA HLA-A*01:01
NetMHCStab HLA-A*01:01
NetMHCstabpan HLA-A*01:01
TLStab HLA-A*01:01
NetMHCpan4.1BA HLA-A*02:01
NetMHCstab HLA-A*02:01
NetMHCstabpan HLA-A*02:01
TLStab HLA-A*02:01
NetMHCpan4.1BA HLA-A*03:01
NetMHCstab HLA-A*03:01
NetMHCstabpan HLA-A*03:01
TLStab HLA-A*03:01
NetMHCpan4.1BA HLA-A*11:01
NetMHCstab HLA-A*11:01
NetMHCstabpan HLA-A*11:01
TLStab HLA-A*11:01
NetMHCpan4.1BA HLA-A*24:02
NetMHCstab HLA-A*24:02
NetMHCstabpan HLA-A*24:02
TLStab HLA-A*24:02
NetMHCpan4.1BA HLA-A*26:01
NetMHCstab HLA-A*26:01
NetMHCstabpan HLA-A*26:01
TLStab HLA-A*26:01
NetMHCpan4.1BA HLA-B*07:02
NetMHCstab HLA-B*07:02
NetMHCstabpan HLA-B*07:02
TLStab HLA-B*07:02
NetMHCpan4.1BA HLA-B*15:01
NetMHCstab HLA-B*15:01
NetMHCstabpan HLA-B*15:01
TLStab HLA-B*15:01
NetMHCpan4.1BA HLA-B*35:01
NetMHCstab HLA-B*35:01
NetMHCstabpan HLA-B*35:01
TLStab HLA-B*35:01
NetMHCpan4.1BA HLA-B*40:01
NetMHCstab HLA-B*40:01
NetMHCstabpan HLA-B*40:01
TLStab HLA-B*40:01
bits
position
